# Integrated analysis of dermal blister fluid proteomics and genome-wide skin gene expression in systemic sclerosis: an observational study

**DOI:** 10.1016/S2665-9913(22)00094-7

**Published:** 2022-06-23

**Authors:** Kristina E N Clark, Eszter Csomor, Corrado Campochiaro, Nicholas Galwey, Katherine Nevin, Mary A Morse, Yee Voan Teo, Johannes Freudenberg, Voon H Ong, Emma Derrett-Smith, Nicolas Wisniacki, Shaun M Flint, Christopher P Denton

**Affiliations:** a Centre for Rheumatology, University College London, London, UK; b Immunoinflammation, GlaxoSmithKline, Stevenage, UK; c Computational Biology, GlaxoSmithKline, California, USA

## Abstract

**Background:**

Skin fibrosis is a hallmark feature of systemic sclerosis. Skin biopsy transcriptomics and blister fluid proteomics give insight into the local environment of the skin. We have integrated these modalities with the aim of developing a surrogate for the modified Rodnan skin score (mRSS), using candidate genes and proteins from the skin and blister fluid as anchors to identify key analytes in the plasma.

**Methods:**

In this single-centre, prospective observational study at the Royal Free Campus, University College London, London, UK, transcriptional and proteomic analyses of blood and skin were performed in a cohort of patients with systemic sclerosis (n=52) and healthy controls (n=16). Weighted gene co-expression network analysis was used to explore the association of skin transcriptomics data, clinical traits, and blister fluid proteomic results. Candidate hub analytes were identified as those present in both blister and skin gene sets (modules), and which correlated with plasma (module membership >0·7 and gene significance >0·6). Hub analytes were confirmed using RNA transcript data obtained from skin biopsy samples from patients with early diffuse cutaneous systemic sclerosis at 12 months.

**Findings:**

We identified three modules in the skin, and two in blister fluid, which correlated with a diagnosis of early diffuse cutaneous systemic sclerosis. From these modules, 11 key hub analytes were identified, present in both skin and blister fluid modules, whose transcript and protein levels correlated with plasma protein concentrations, mRSS, and showed statistically significant correlation on repeat transcriptomic samples taken at 12 months. Multivariate analysis identified four plasma analytes as correlates of mRSS (COL4A1, COMP, SPON1, and TNC), which can be used to differentiate disease subtype.

**Interpretation:**

This unbiased approach has identified potential biological candidates that might be drivers of local skin pathogenesis in systemic sclerosis. By focusing on measurable analytes in the plasma, we generated a promising composite plasma protein biomarker that could be used for assessment of skin severity, case stratification, and as a potential outcome measure for clinical trials and practice. Once fully validated, the biomarker score could replace a clinical score such as the mRSS, which carries substantial variability.

**Funding:**

GlaxoSmithKline and UK Medical Research Council.

## Introduction

Examination of the skin of patients with systemic sclerosis provides insight into the biology of fibrosis that might be relevant to other organs and diseases. Skin severity in systemic sclerosis can be assessed using the modified Rodnan skin score (mRSS)^1^ in clinical practice and trials.^2^ However, the mRSS is limited by inter-operator variability and poor understanding of the link between clinical measurement and relevant disease biology.^3^ Although direct sampling of lesional tissue by skin biopsy can be performed, there are drawbacks, including cosmetic scarring, risk of infection, and inconvenience of the procedure. Less invasive approaches are attractive, especially blood sampling, but the results are difficult to interpret in a multicompartment disease such as systemic sclerosis. A biochemical surrogate based upon gene or protein expression that is reliable, reproducible, and allows for improved monitoring is therefore desirable.

Gene expression transcriptomic signatures of the skin have now been well defined in systemic sclerosis.^4^, ^5^, ^6^ However, findings do not always correlate with protein expression or function within the skin. Proteins can be directly examined in skin biopsies or in dermal suction blister fluid, giving insight into the local environment in cells. Proteomic analysis offers the potential to improve interpretation of gene expression. Because interstitial fluid might also reflect plasma protein levels we hypothesised that dermal blister fluid protein analysis could validate gene expression differences in biopsies and help to identify plasma proteins that have altered expression within the dermal microenvironment.

**Research in context**

**Evidence before this study**

Systemic sclerosis is a heterogenous disease, whereby current tools for assessing skin involvement such as the modified Rodnan skin score (mRSS) are limited by inter-observer variability. We searched PubMed up to Sept 1, 2021, using the terms “systemic sclerosis”, “scleroderma”, “skin score” and “biomarker” without language restriction. Despite some success identifying RNA transcripts that reflect patient subgroups and skin severity, there is currently no available biomarker in the plasma that accurately reflects the local molecular environment of the skin.

**Added value to this study**

This prospective study used integrated analysis of transcriptomics from skin biopsies and proteomics from blister fluid to identify key analytes present in both RNA transcripts and proteins in the interstitial fluid that correlate with degree of skin involvement in systemic sclerosis, and correlate with protein levels in the blood. Multivariate analysis confirmed four of these analytes were statistically significant and independently correlated to skin involvement. These were COL4A1, COMP, TNC, SPON1. Interestingly, each of these candidate markers might reflect distinct facets of skin pathogenesis and so add substantial value to previous studies independently linking these proteins to mRSS.

**Implications of all the available evidence**

Our results highlight the use of skin blister fluid as an anchor for deriving key analytes that are also measurable in the plasma and reflect skin severity. In a multicompartment disease, it is important to define potential biomarkers of discrete manifestations such as skin rather than conflating burden from multiple organ involvement.

To explore this hypothesis, we sampled dermal interstitial fluid and then extrapolated our findings to plasma. Our method of linking plasma levels to local gene expression and interstitial fluid protein mitigates the limitation of systemic sampling in a multicompartment disease.^7^

Weighted gene coexpression network analysis (WGCNA) is a systems biology method used to explore characteristics of gene networks related to complex diseases and to investigate association between genome and clinical features and identify candidate biomarkers.^8^, ^9^ We have used WGCNA to link transcriptomic data with skin gene expression and dermal interstitial fluid protein expression and anchored this to clinical traits to identify key modules, thus allowing us to identify gene and protein combinations which best associate with skin disease severity and use this to develop a potential composite plasma surrogate for mRSS.

## Methods

### Study design and patients

This was a single-centre, prospective observational study at the Royal Free Campus, University College London, London, UK, comprising of four distinct participant cohorts: patients with early diffuse cutaneous systemic sclerosis (dcSSc; <5 years disease duration), late dcSSc (>5 years disease duration), limited cutaneous systemic sclerosis (lcSSc), and healthy controls. This study received ethical approval from the NHS Research and Ethics Committee (REC number 6398). All participants provided written informed consent for their participation.

Patients with systemic sclerosis that fulfilled the 2013 American College of Rheumatology-European League Against Rheumatism classification criteria^10^ and diffuse or limited subsets according to LeRoy and colleagues^11^ were included in the study.

The biological phenotyping of diffuse systemic sclerosis (BIOPSY) study is outlined in the appendix (pp 2–3) and by Clark and colleagues.^5^ All patients in the BIOPSY study were included in the cross-sectional analysis, with a prospective cohort of patients with early dcSSc followed up every 3 months for 12 months. Patients were recruited from our tertiary connective tissue disease centre between September, 2016, and April, 2018.

The study was designed to recruit a feasible and representative discovery cohort of patients with systemic sclerosis, with the goal of including at least six patients for each major disease or autoantibody subgroup, and at least 20 patients with early dcSSc.

### Procedures

For all participants, serum, plasma, PAXgene Blood RNA tubes (Qiagen, Hilden, Germany), suction blister fluid, and skin biopsy samples were all collected on the same day.

Skin biopsies (4 mm) were obtained from the forearm of participants, collected into RNAlater stabilisation solution (Qiagen, Hilden, Germany) and stored at –80°C. For patients with dcSSc, the biopsy was taken from involved skin, at least 3 cm from previous biopsies.

Forearm skin blister fluid was obtained from the opposite arm, using the dermal suction blister method,^5^ left for 2·5 h, with a suction pressure 280 mm Hg, and stored at −80°C.

Plasma and blister samples were assayed using the Olink multiplex platform (Olink Proteomics AB, Uppsala, Sweden) for proteomic analysis (proteins), which is based on Proximity Extension assay technology. This allowed for the reporting of normalised protein expression, corresponding to log2 (expression).

RNA transcript analysis was performed on skin samples stored in RNAlater stabilisation solution and blood samples collected and stored in PAXgene Blood RNA tubes. RNA expression analysis was carried out by Epistem, Manchester, UK. RNA was isolated in two batches per tissue type, and the RNA was sequenced in batches of 20 samples on the Illumina NextSeq 550 (Illumina, San Diego, CA, USA).

### Statistical analysis

Statistical analysis was carried out using the software R (version 3.6.0). Statistical workflow is shown in appendix (p 5). WGCNA was analysed utilising the WGCNA R packages. All genes and proteins were included to construct the modules using the WGCNA algorithms. A module is a cluster of densely interconnected genes in terms of coexpression. WGCNA constructs a scale-free network by correlating RNA transcript concentrations with clinical feature. The minimum number of analytes in each module was 30, and each module was allocated a random colour title.

The blister and skin modules that had a statistically significant correlation to a diagnosis of systemic sclerosis and a statistically significant correlation to the other tissue modality (eg, a blister module had to both correlate to dcSSc diagnosis, and to the skin module that correlated to dcSSc diagnosis), were selected for further analysis. This process was repeated for plasma proteomics and blood transcriptomics.

Spearman rank was used to calculate correlations, and ANOVA with Tukey post-hoc analysis was used to calculate the difference in expression of analytes between patient groups where appropriate. The Benjamini-Hochberg false discovery rate was used for multiple comparisons across the study, with a threshold of significance of less than 0·05. We defined the gene significance as the correlation coefficient between individual genes and the biological trait of early dcSSc. Module membership quantifies how close a gene is to a given module. Hub analytes were selected based on a gene significance of more than 0·6, a module membership of more than 0·7, and p<0·010. The hub analytes that met these criteria in both the blister fluid, and in the skin transcriptomic modules were selected for further analysis.

The RNA transcripts obtained from skin biopsy samples from patients with early dcSSc at 12 months were used for confirmation of the hub analytes (17 genes). Statistically significant correlation between transcripts and mRSS were used to reinforce the significance of the selected potential candidates for composite biomarker development.

Development of a candidate composite biomarker was performed based upon plasma variables identified from hub analytes. Multivariable analysis was performed using least absolute shrinkage and selection operator (LASSO) regression models^12^ in the glmnet package. The ordinary least squares regression model was used to estimate the relationship between predicted and actual mRSS.

### Role of the funding source

The study was designed in collaboration between both the funders and University College London. The funders of the study had no role in the collection of samples or data collection. Data analysis was primarily carried out by University College London, with guidance from the statistical team at GlaxoSmithKline. The funders were involved in data interpretation and writing of the report.

## Results

The BIOPSY data set was generated for integrated analysis of skin and blood samples collected from 52 patients with systemic sclerosis, and 16 sex-matched healthy controls. Cohort characteristics of the 52 patients in the BIOPSY data set have previously been described^5^ and are in the appendix (p 4). 36 (69%) of 52 patients with systemic sclerosis were women and 16 (31%) were men (appendix p 4); median disease duration was 1·8 years (IQR 1·0–2·6) in the early dcSSc cohort (n=21), 13·0 years (8·0–17·8) in the late dcSSc cohort (n=15), and 9 years (5·2–14·4) in the lcSSc cohort (n=16). 48 (92%) of 52 patients had antinuclear antibodies, of whom 14 (27%) had anti-topoisomerase antibodies, 12 (23%) had anti-RNA polymerase III antibodies, and 25 (50%) had other antibodies. Median baseline mRSS was 18·0 (IQR 11·0–32·5) for patients with early dcSSc, 10·0 (5·0–14·0) for those with late dcSSc, and 4·0 (3·0–4·5) for lcSSc (appendix p 4). One systemic sclerosis-related death occurred during follow up. Immunosuppression in patients with early dcSSc at baseline included mycophenolate mofetil (nine [43%] of 21) and methotrexate (seven [33%]). Blister fluid and paired plasma samples were available for 41 (79%) of 52 patients with systemic sclerosis, and all healthy controls (table).

**Table:** Demographics of participants and samples included in proteomics analysis

		**Early dcSSc at baseline (n=14)**	**Late dcSSc (n=11)**	**LcSSc (n=16)**	**Healthy controls (n=16)**
Sex					
	Female	7 (50%)	9 (82%)	12 (75%)	9 (56%)
	Male	7 (50%)	2 (18%)	4 (25%)	7 (44%)
Age, years		51·0 (32·0–64·0)	56·9 (46·0–65·0)	52·5 (47·5–61·0)	43·3 (31·8–48·8)
Disease duration, years		2·0 (1·3–2·6)	11·5 (8·0–17·8)	9·0 (5·2–14·4)	..
Modified Rodnan skin score		17·0 (11·0–27·0)	11·0 (4·0–16·0)	4 ·0 (3·0–4·5)	..
Antibody					
	Anti-topoisomerase antibody	3 (21%)	2 (18%)	2 (13%)	..
	Anti-RNA polymerase III antibody	4 (29%)	6 (55%)	0	..
	Anti-centromere antibody	0	0	10 (63%)	..
	Antinuclear antibody negative	2 (14%)	1 (9%)	1 (6%)	..
	Other	5 (36%)	2 (18%)	3 (19%)	..
Organ involvement					
	Lung	3 (21%)	5 (45%)	0	..
	Muscle	4 (29%)	1 (9%)	0	..
	Kidney	3 (21%)	1 (9%)	0	..
	Pulmonary arterial hypertension	1 (7%)	1 (9%)	0	..
	Cardiac	3 (21%)	1 (9%)	1 (6%)	..
	Gastrointestinal	1 (7%)	4 (36%)	1 (6%)	..
Overlap conditions					
	Rheumatoid arthritis	1 (7%)	0	1 (6%)	..
	Polymyositis or dermatomyositis	4 (29%)	3 (27%)	0	..
	Sjögren's syndrome	0	1 (9%)	2 (13%)	..
Immunosuppression at time of sample collection					
	Mycophenolate mofetil	6 (43%)	7 (64%)	0	..
	Methotrexate	4 (29%)	2 (18%)	3 (19%)	..
	Hydroxychloroquine	3 (21%)	1 (9%)	5 (31%)	..
	Azathioprine	1 (7%)	0	0	..
	Tocilizumab	1 (7%)	0	0	..
	Cyclophosphamide	1 (7%)	0	0	..
	Intravenous immunoglobulin	0	1 (9%)	0	..
	Untreated	2 (14%)	3 (27%)	9 (56%)	..

Data are n (%) and median (IQR). dcSSc=diffuse cutaneous systemic sclerosis. LcSSc=limited cutaneous systemic sclerosis.

Pre-processing and normalisation of the transcript data were performed as per our previous published work.^5^ Normalised fragments per kilobase of transcripts per million values were obtained using rlog() function within DESeq2. In total, 58 884 gene transcripts were included for transcriptional analysis, and 1196 proteins from proteomic analysis.

**Figure 1:**
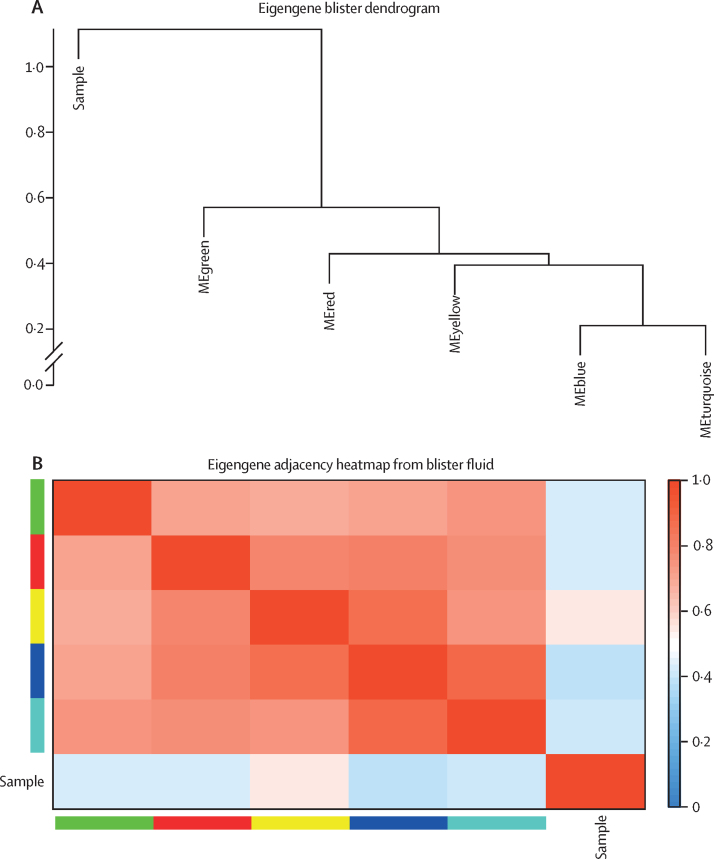

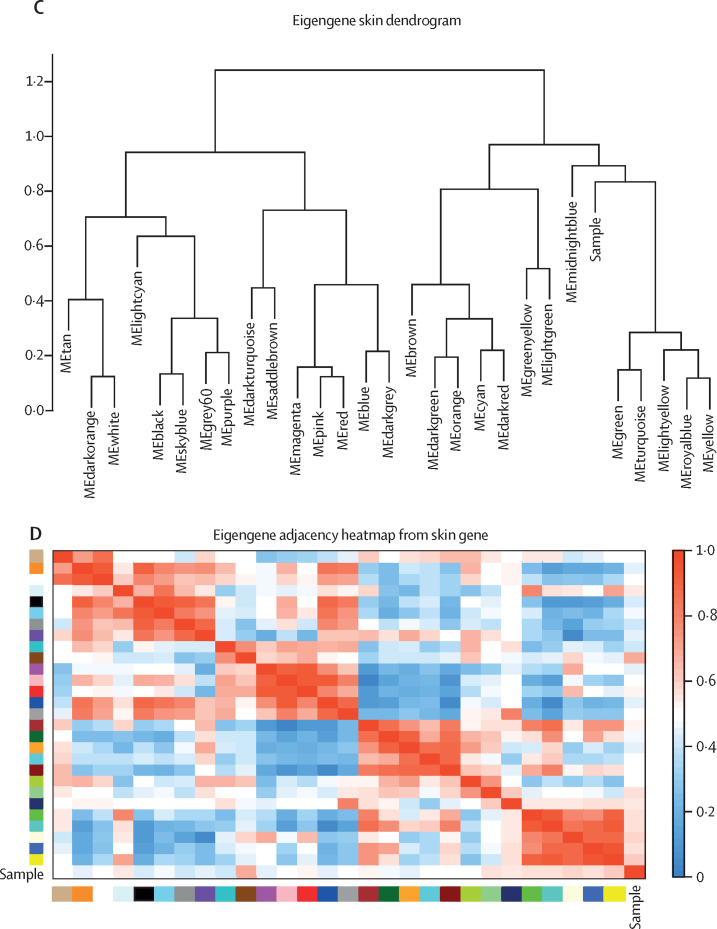

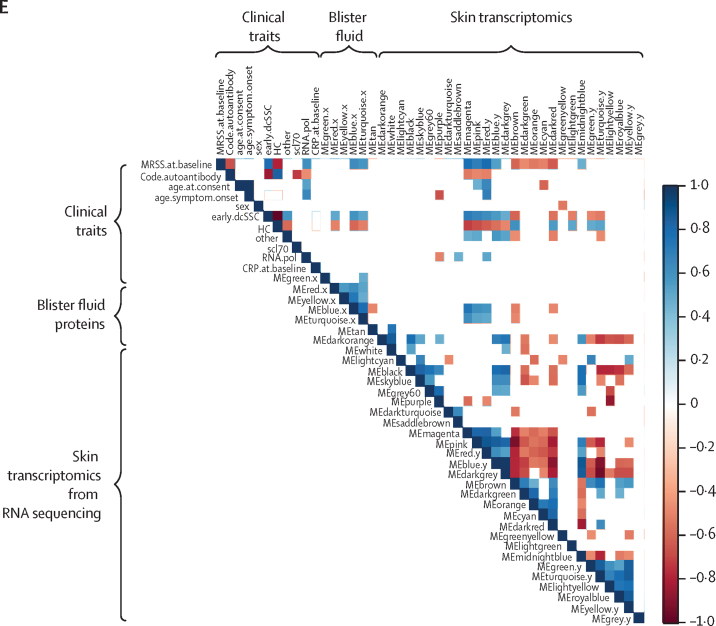
Construction of weighted gene coexpression network analysis and identification of significant modules (A) Clustering dendrogram of blister fluid module eigengenes. (B) Heatmap plots of the eigengene adjacencies in blister fluid modules. Each coloured row and column in the heatmap corresponds to one module eigengene. (C) Clustering dendrogram of gene expression module eigengenes from skin. (D) Heatmap plots of the eigengene adjacencies in skin gene expression modules. Each coloured row and column in the heatmap corresponds to one module eigengene. (E) Correlation matrix highlighting only significant correlations with p<0·05 between clinical traits, blister fluid proteomics, and skin transcriptomics. Blue indicates a positive correlation and a red negative correlation. x and y are used to differentiate modules originating from different tissues. CRP=C-reactive protein. dcSSC=diffuse cutaneous systemic sclerosis. HC=healthy control. ME=module eigengene. mRSS=modified Rodnan skin score. RNApol=anti-RNA polymerase III. Scl70=anti-topoisomerase antibody.

First, a scale-free topology model was constructed, using the soft-thresholding power of 7·0 for blood and skin, and standard deviation parameter for soft-thresholding of 0·2 for blister fluid and 0·3 for plasma (appendix p 6). A total of 28 modules were identified in the skin, five modules in the blister fluid (figure 1A and 1C), five modules in plasma, and 36 modules in blood transcripts by hierarchical clustering of analyte expression and dynamic tree cut method. The eigengene adjacency heatmap revealed the interaction relationships between the modules in each tissue subtype (figure 1B and 1D; appendix pp 6, 15).

Correlation matrices between clinical traits and the modules were created. In the skin, five key modules (magenta, pink, red, blue, and dark turquoise) significantly correlated with clinical trait of early dcSSc (p<0·050; appendix p 7), as well as five modules in the blister fluid (blue, turquoise, red, yellow, and grey; p<0·050; appendix p 7). Focusing on results from blister fluid and skin transcriptomics, modules were selected that correlated with a diagnosis of early dcSSc, and a statistically significant correlation between skin and blister fluid modules. The blue and turquoise modules in blister fluid, and the magenta, pink, and red modules in the skin were the only modules that met these criteria (figure 1E). The mean gene significance in each module (correlation of each gene within module to a particular clinical trait) all positively correlated to a diagnosis of early dcSSc (appendix p 8). The number of genes in each skin module were: magenta (385 genes), red (446 genes), and pink (411 genes). The number of proteins in each significant blister module were: blue (450 proteins) and turquoise (370 proteins). The highest correlation between module membership and gene significance to early dcSSc was in the blue module (*r*=0·62, p<0·0001) in blister fluid, and with the red module (*r*=0·84, p<0·0001) in skin gene expression.

Each module was subjected to further analysis of their biological processes. Within skin gene expression modules there were three main themes: extracellular matrix organisation (magenta), adaptive immunity (pink), and interferon signatures (red). Within the blister fluid, the main themes were focused on metabolic processes (turquoise), and extracellular matrix organisation and cell interaction (blue; appendix pp 9–10). Overlapping Hallmark pathways showed differing normalised enrichment scores between modules (appendix p 10).

**Figure 2:**
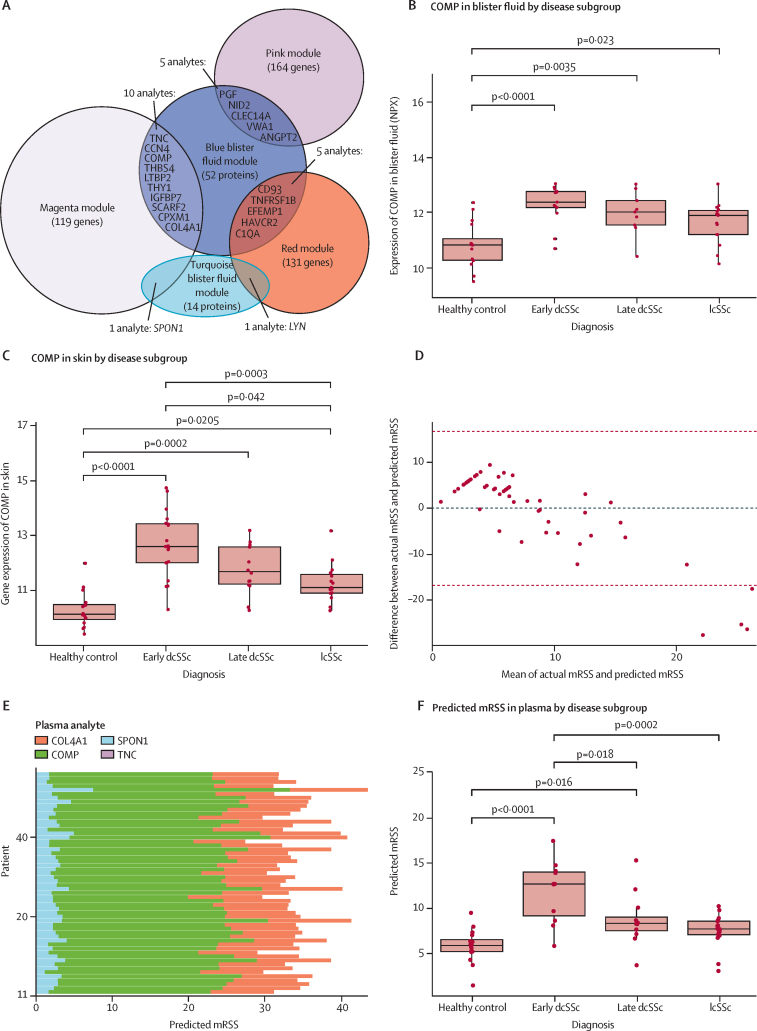
Identification of hub genes and application to potential prediction model (A) Venn diagram showing number of hub genes in each module according to criteria of module membership >0·7, and gene significance >0·6 and p<0·010. Listed analytes identified as a hub analyte in skin module and blister fluid module. (B) Box and whisker plots with ANOVA results from COMP analysis across disease spectrum in blister fluid. (C) Box and whisker plots with ANOVA results from COMP analysis across disease spectrum in skin gene expression. (D) Bland-Altman plot of predicted and actual mRSS. (E) Contribution of each plasma analyte to predicted mRSS across the whole systemic sclerosis spectrum. (F) Predicted mRSS in each systemic sclerosis subgroup and healthy controls. Only significant post-hoc p values are included. dcSSc=diffuse cutaneous systemic sclerosis. lcSSc=limited cutaneous systemic sclerosis. mRSS=modified Rodnan skin score. NPX=normalised protein expression.

The analytes in each module that had both a high module membership (>0·7, p<0·010), and a strong correlation to early dcSSc (gene significance >0·6, p<0·010) were defined as hub analytes (130 magenta genes, 137 red genes, 169 pink genes, 72 blue proteins, and 16 turquoise proteins). 22 of these hub analytes were present in both blister modules and skin modules (figure 2A). Example plots for one analyte (cartilage oligomatrix protein [COMP]) in blister and skin are shown in figure 2B and 2C.

An ideal analyte is one identified in plasma as a marker of skin disease, and applicable across the whole systemic sclerosis spectrum. We first confirmed that the transcriptional results of these 22 analytes in the skin correlated with their protein concentration in the blister fluid across the whole systemic sclerosis spectrum at baseline (appendix p 11). We then looked for statistically significant correlation between the blister fluid analyte concentrations and plasma fluid concentrations. 19 of the analytes met these criteria. None of the analytes showed statistical correlation between blood transcriptomics and plasma protein concentrations. There was a high degree of correlation between skin transcripts and plasma proteins.

**Figure 3:**
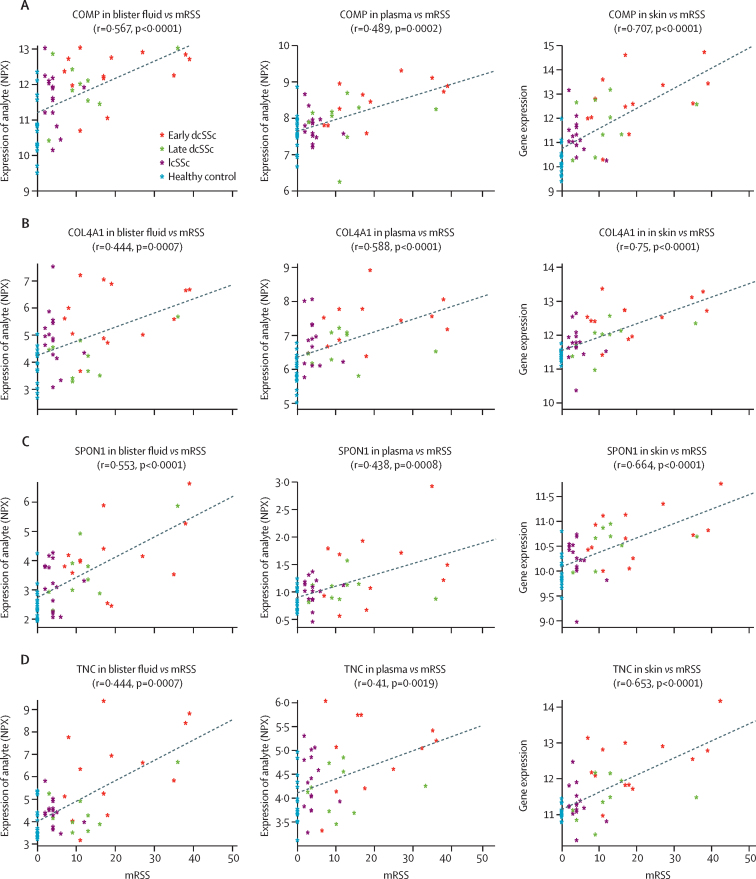
Scatter plots of four analytes expression values identified in our prediction model against mRSS across tissue types (blister fluid, plasma, and skin) Correlation coefficient and p value included for COMP (A), COL4A1 (B), SPON1 (C), and TNC (D). dcSSc=diffuse cutaneous systemic sclerosis. lcSSc=limited cutaneous systemic sclerosis. mRSS=modified Rodnan skin score. NPX=normalised protein expression.

All 19 analytes in skin transcriptomics and blister protein concentrations correlated significantly to mRSS across the disease spectrum. Within the plasma protein concentrations, 17 analytes showed this statistical correlation (p<0·050; figure 3; appendix pp 11–12).

We used the skin transcriptomics from 12-month skin biopsy samples to extend the analysis, to test whether there was still a correlation between mRSS and the 17 analytes at a different time point. This would suggest the analysis is sensitive to a change over time, and potentially be extrapolated to plasma. Performing this analysis, 11 analytes correlated significantly with mRSS at 12-month biopsies (appendix p 11). These were ANGPT2, CCN4, CD93, CLEC14A, COL4A1, COMP, SCARF2, SPON1, THBS4, THY1, and TNC. All these 11 plasma analytes were significantly different by disease subgroup (appendix pp 13–14).

To obtain a parsimonious predictor of mRSS, we performed a LASSO multiple regression on the plasma protein expression values for the 22 hub analytes identified as being present in both blister fluid modules and skin transcript modules, and fulfilling the criteria of having a module membership of more than 0·7, and a gene significance of more than 0·6. This analysis identified four analytes that were predictive of mRSS. These analytes were COL4A1, COMP, SPON1, and TNC. Our prediction model identified the following equation for mRSS assessment using plasma variables:

mRSS= – 25·898 + 0·0014(TNC) + 2·818(COMP) + 1·343(COL4A1) + 2·562(SPON1)

The ordinary least squares regression model showed the model to be significant with *r*=0.617, p=0·022. The Bland-Altman plot shows better conformity of results for mRSS less than 20 (limits of agreement 16·8 to –16·8; figure 2D), with a statistically significant correlation between predicted and actual mRSS (*r*=0·614, p<0·0001). The relative weights of each analyte show the variability between samples in predicting the skin score (figure 2E) and the predicted mRSS was statistically higher in early dcSSc compared with other systemic sclerosis subgroups on ANOVA (figure 2F). These four plasma analytes also showed a strong correlation with their blister proteomic expression, and a strong correlation with mRSS (appendix p 11).

Similar analysis was attempted on integrating plasma proteomics, blood transcriptomics and clinical traits. One plasma module (turquoise), and one blood module (skyblue2) correlated with a diagnosis of early dcSSc. Functional annotation was not able to be performed, as none of the modules reached significance. Therefore, no further analysis was performed using the blood transcriptomics data (appendix p 15).

## Discussion

Recent observational cohort studies and clinical trials have highlighted clinical diversity in systemic sclerosis. There is a need for better biomarkers for stratification, classification, diagnosis, and management, as well as to aid assessment of treatment response. This need is illustrated by the limitations of mRSS in clinical trials.^13^, ^14^ Whereas previous studies have developed markers for longitudinal assessment,^15^ the present work has explored biological differences in plasma protein and gene expression that directly reflect pathobiology of skin.

Our previous work has shown that skin blister fluid samples reflect the local environment of the skin cells.^16^ Because plasma proteomics and RNA transcripts from blood do not necessarily correlate,^7^, ^17^ we have used blister proteomics as an anchor to identify analytes in the skin that correlate with skin severity, and that can also be measured in plasma. Our analysis selected four key plasma analytes (COL4A1, COMP, SPON1, and TNC), as potential predictors of mRSS.

The fibrillar collagen molecule COL4A1, is mainly found at the dermo-epidermal junction in the skin and in the basement membrane surrounding blood vessels and sweat glands. COL4A1 is synthesised by endothelial cells and pericytes and its primary function is in angiogenesis.^18^ COL4A1 has an established role in other fibrotic diseases including liver fibrosis, Goodpasture's syndrome, and Alport syndrome. Mouse models relevant to systemic sclerosis, have confirmed increased gene expression of *COL4A1* in the skin and lung.^19^ Serum COL4 is found more abundantly in patients with systemic sclerosis, especially those with early dcSSc, compared with healthy controls, with serum levels positively correlating with mRSS.^7^, ^18^ Johnson and colleagues reported that *COL4A1* was one of the key upregulated genes in their inflammatory subset of patients with systemic sclerosis.^6^

COMP is a matricellular protein regulated by TGFβ and is involved in the assembly and maintenance of the fibrillar collagen extracellular network.^20^ Serum concentrations of COMP are elevated in patients with systemic sclerosis (dcSSc more than lcSSc) and correlate with mRSS.^21^ Elevated COMP concentrations are also associated with severe internal organ involvement^22^ and act as predictors of mortality in early disease. More specifically, mRNA expression of COMP is seen in systemic sclerosis fibroblasts from the skin.^23^ In combination with THBS1, and IFN-regulated genes (*SIGLEC1* and *IFI44*), *COMP* expression was a validated predictor of mRSS cross-sectionally.^15^, ^20^

*SPON1* is the gene coding for spondin-1, which is an extracellular matrix glycoprotein. Spondins themselves are well-conserved extracellular matrix proteins characterised by the presence of thrombospondin domains. *SPON1* is a key component of the WNT protein pathway and acts as an adhesion molecule in the basement membrane. Serum concentrations are found to be elevated in systemic sclerosis compared to healthy controls, and further upregulated by adiponectin.^24^ In one longitudinal proteomic study, SPON1 was one of two analytes which best described longitudinal change in mRSS (the other being ST2 [IL1R4]).^25^

TNC, another extracellular matrix protein induced by TGFβ, is the best studied endogenous toll-like receptor 4 ligand. Although usually undetectable in healthy adult tissue, it is highly upregulated in systemic sclerosis skin biopsies.^26^ TNC is the chief damage-associated molecular patterns activating resident fibroblasts through toll-like receptor 4,^27^ and promoting differentiation of myofibroblasts. Mice without TNC were protected from fibrosis.^27^ Serum concentrations of TNC are elevated in early and late stage systemic sclerosis,^7^, ^27^ and correlate with mRSS.^28^ More recently, *TNC* was identified as one of ten hub differentially expressed genes in skin that could sensitively and specifically distinguish systemic sclerosis from healthy controls.^29^

WGCNA has previously brought insight to other autoimmune diseases such as rheumatoid arthritis,^8^ and in systemic sclerosis to identify key hub genes associated with clinical traits such as pulmonary arterial hypertension,^9^ as well as potential drug targets. To our knowledge, this is the first time that gene expression and proteomic modules have been integrated to identify key analytes.

Plasma or serum markers are attractive predictive variables due to ease of measurement and patient acceptability. This novel approach identified analytes in the plasma which correlate with the extent of skin fibrosis and assessed their contribution using a multiple regression model. Thus, our overarching study concept was to use skin transcriptomics and blister fluid protein expression data to identify candidate proteins for a composite plasma marker of mRSS that reflects relevant skin biology in systemic sclerosis pathogenesis.

Individually, the four key analytes have not proved robust enough to predict both skin disease at baseline and change over time. Studies focussing on extracellular matrix turnover markers, have looked promising, although their use is not always clear.^30^ In the future, plasma analysis can be used for further validation and longitudinal studies to ask if we can use similar strategies or the same composite as a pharmacodynamic biomarker for mRSS as proposed in previous work.^15^ There is a need for a similar composite biomarker assessment in the blood in systemic sclerosis, which accurately assesses skin disease. Once validated, our composite marker has the potential to be used in this way.

Our study has several strengths. It is the first attempt to use an integrated approach of both proteomics and transcriptomics to identify key analytes that can be used to diagnose extent and severity of skin involvement. The blister fluid allowed for a unique opportunity to sample the local environment and put the gene expression results into context. Our work is also strengthened by the broad spectrum of patients with systemic sclerosis included, allowing interpretation of the results across the disease spectrum. The fact that this study has a treated cohort of patients, means that interpreting the data will be relevant to current clinical practice. We have already shown that the early dcSSc cohort's skin trajectories were not confounded by immunosuppression.^5^ Furthermore, clinical trials are now generally designed to permit background therapy, which makes our approach highly relevant.

There are also some limitations. This is a single centre study, with a relatively small cohort of well-defined patients. The ability to create a blister in patients was not of uniform ease, and technical issues resulted in insufficient blister fluid for some patients despite adequate suction and time. This has resulted in a larger discrepancy between the healthy controls and early dcSSc in terms of sex and age than is seen across the whole cohort, with potential bias being introduced. Additionally, although the number of proteins assessed in the Olink panels is one of the largest done on a cohort of patients, there might have been some pertinent proteins that were not analysed. However, with careful collaboration with Olink, panels were selected to optimise the full range of candidate biomarkers to be included. Because plasma proteomes were not available for the 12-month samples, it was not possible to examine the relationship between change in proteins and mRSS. Future work would also allow for exploration of a relationship with forced vital capacity, not deemed informative in this present study due to the relative stability of lung function in patients with plasma analytes available.

We have used an integrated analytical approach to interrogate high dimensional data from a well characterised cohort of patients with systemic sclerosis receiving standard of care treatment. By using local gene expression and protein analysis in skin blister fluid we have identified a small number of candidate plasma proteins that could be easily incorporated into an accessible composite biomarker, and potentially replace mRSS in the clinical setting. Our preliminary studies support further work to refine and develop such a marker that would better reflect disease biology in systemic sclerosis and benefit clinical practice, research, and interventional trials.

## Data sharing

All data, code, and materials used in the analysis are available upon request for the purposes of reproducing or extending the analysis via the corresponding author, in accordance with local and institutional guidance and legal requirements.

## Declaration of interests

CPD reports consulting fees or honoraria from Janssen, GlaxoSmithKline, Roche, Boehringer Ingelheim, Sanofi, Galapagos, Inventiva, Corbus, Acceleron, Horizon, Gesynta, and ARXX Therapeutics; and from research grants to their institution from GlaxoSmithKline, ARXX Therapeutics, Servier, and Horizon Therapeutics. NW, SMF, YVT, JF, NG, EC, KN, and MAM are employees of GlaxoSmithKline. NG, EC, JF, NW, MAM, SMF, and KN are shareholders in GlaxoSmithKline. All other authors declare no competing interests
