# Supplementary material for: Integrated analysis of dermal blister fluid proteomics and genome-wide skin gene expression in systemic sclerosis: an observational study
Source: Lancet Rheumatol. 2022 Jun 23;4(7):e507–16. doi: 10.1016/S2665-9913(22)00094-7 (PMC9669928; doi:10.1016/S2665-9913(22)00094-7)

THE LANCET
Rheumatology

Supplementary appendix

This appendix formed part of the original submission and has been peer reviewed. We post it as supplied by the authors.

Supplement to: Clark KEN, Csomor E, Campochiaro C, et al. Integrated analysis of dermal blister fluid proteomics and genome-wide skin gene expression in systemic sclerosis: an observational study. *Lancet Rheumatol* 2022; **4:** e507–16.

Appendix

Supplementary Methods

Sample collection

Details regarding serum markers and histology are beyond the scope of this study, but are included in the published manuscript by Clark et al[5].

Informed consent was obtained from all subjects. Consent included the use of their clinical data and samples for research purposes, as well as for any minor risks associated with skin biopsy or blister including infection.

The early dcSSc cohort were reviewed every 3 months over a 12-month period, with clinical assessment and sample collection at that time.

MRSS was assessed at the time of sample collection by one of two well-trained and experienced observers with consistent scoring techniques.

Plasma collection

Plasma was also isolated (centrifuged at 2000xg at 4°C for 15 minutes) and aliquoted into 400 µl samples. These were then stored at -80°C.

Data collection

Organ involvement was defined as follows: interstitial lung disease- >20% involvement on HRCT, polymyositis confirmed with CK and/or electromyography, and renal involvement by history of previous renal crisis. PAH was confirmed with right hear catheter, cardiac involvement as confirmed with cardiac MRI, and GI involvement was the requirement of more than 3 courses of antibiotics a year for bacterial overgrowth, confirmed GAVE on endoscopy, the need for parenteral nutrition, or intervention for SSc anorectal disease.

Bias

To reduce bias and confounders between HC and early dcSSc, patients were recruited across the whole SSc spectrum, and allowed differences between HC and early dcSSc to be interpreted in the context of later stage disease. This ensured representative patients from all autoantibody subgroups, organ complications and immunosuppressive regimes. The fact clinical assessment was only carried out by two well trained recruiters, with concordant MRSS assessments reduced interobserver variability.

Statistical Analysis using R.software

For the RNAseq results, normalised FPKM (fragments per kilobase of transcript per million) values were obtained using rlog() function within DESeq2 of Rsoftware.

Proteomic analysis was read in NPX values, which are log_2 transformed relative expression values. Analysis was carried out in the log scale. Where the level of response variable was below the threshold of detectability, the lowest detectable level was assigned. Where paired samples were required (e.g. blister and skin analytes for correlation), patients with missing samples were removed from this analysis.

WGCNA (weighted gene coexpression network analysis), was analysed utilising the R packages "WGCNA", as well as "tidyverse", and "hclust".

All genes and proteins were included to construct the modules using the WGCNA algorithms. The soft threshold power was selected according to the standard scale-free networks. The dynamic tree cut method was performed to identify the module by hierarchically clustering genes

Module eigengenes are the first principal component of the module. These were constructed and used to calculate correlation between the modules and clinical traits, utilising Pearson correlation coefficient. Hierarchical clustering by adjacency-based dissimilarity within the eigengene network can then be visualised.

Pathway analysis of biological processes of each module selected was carried out using software R programmes "clusterProfiler", "org.Hs.eg.db", "AnnotationHub" and "DBI". Hallmark pathway enrichment was analysed using programme "msigdbr". Statistically significantly differentially expressed analytes (FDR<0.05) were included in pathway analysis.

Non-parametric analysis utilised the software R programmes "ggplot" and "ggpubr".

Supplementary figure 1: Schematic of BIOPSY cohort and study overview

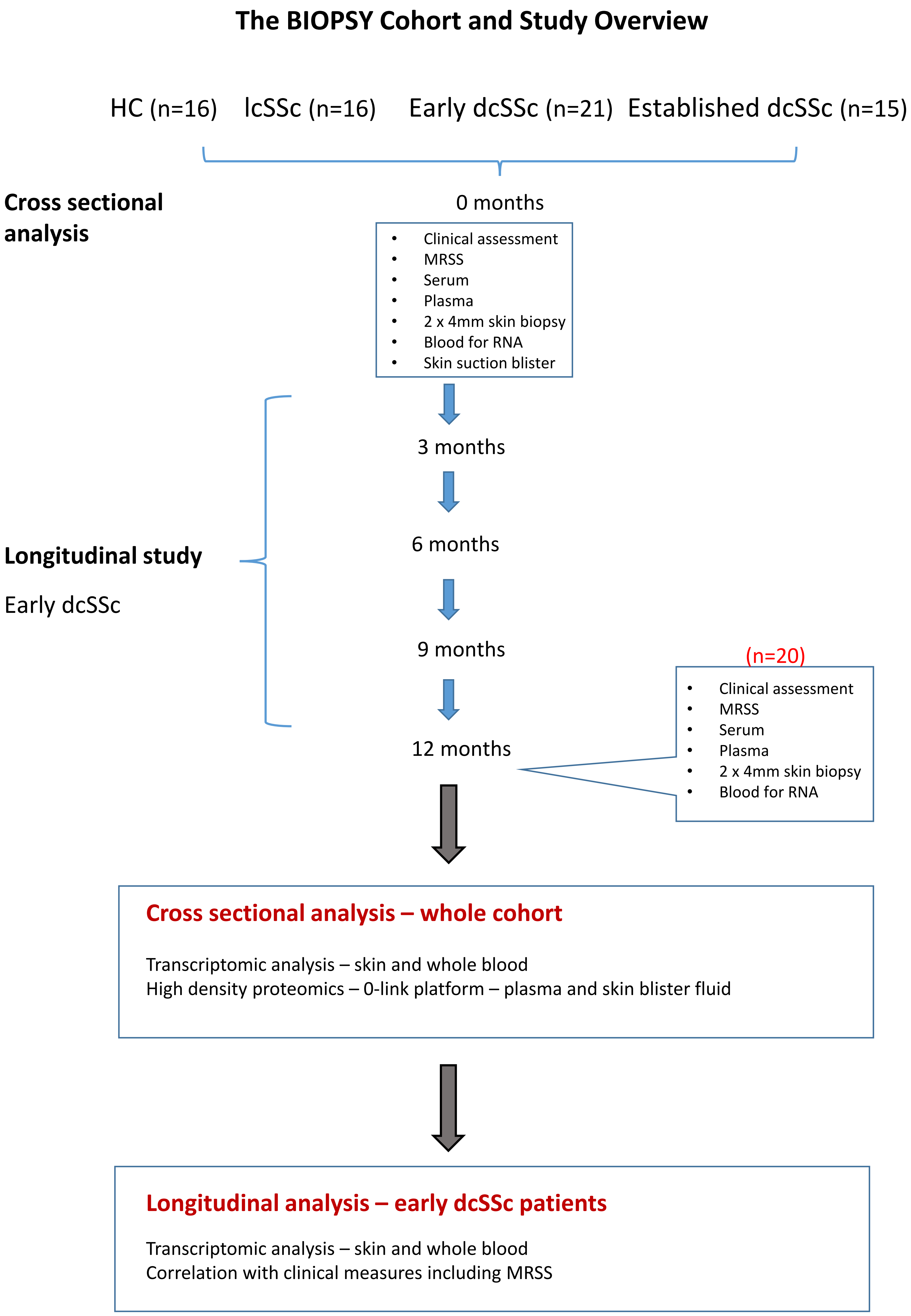

Supplementary Table 2: Demographics of all subjects recruited into BIOPSY cohort at baseline

	early dcSSc at baseline	Late dcSSc	LcSSc	HC
Total (n)	21	15	16	16
F (%)	12 (57.1)	12 (80)	12 (75)	9 (56.3)
Age (yrs)	52 (35.5-66)	56.9 (37-65)	52.5 (47.5-61)	43.3 (31.8-48.8)
Disease duration (yrs)	1.75 (1-2.6)	13 (8-17.8)	9 (5.2-14.4)	
MRSS	18 (11-32.5)	10 (5-14)	4 (3-4.5)	
**Antibody**				
ATA (%)	8 (38.1)	4 (26.7)	2 (12.5)	
ARA (%)	6 (28.6)	6 (40)	0	
ACA (%)	0	0	10 (62.5)	
ANA neg (%)	2 (9.5)	1 (6.7)	1 (6.3)	
Other (%)	5 (23.8)	7 (46.7)	3 (18.8)	
**Organ involvement**				
Lung (%)	6 (28.6)	8 (53.3)	0	
Muscle (%)	6 (28.6)	1 (6.7)	0	
Kidney (%)	3 (14.3)	1 (6.7)	0	
PAH (%)	1 (4.8)	1 (6.7)	0	
Cardiac (%)	3 (14.3)	1 (6.7)	1 (6.3)	
GI (%)	3 (14.3)	4 (26.7)	1 (6.3)	
**Immunosuppression**				
MMF (%)	9 (42.9)	9 (60)	0	
MTX (%)	7 (33.3)	2 (13.3)	3 (18.8)	
HCQ (%)	5 (23.8)	1 (6.7)	5 (31.3)	
Azathioprine (%)	1 (4.8)	0	0	
Tocilizumab (%)	1 (4.8)	0	0	
Cyclophosphamide (%)	1 (4.8)	0	0	
IvIG (%)	0	1 (6.7)	0	
Untreated (%)	3 (14.3)	5 (33.3)	9 (56.2)	

Supplementary figure 2: Schematic to show discovery pipeline of the 4 key analytes

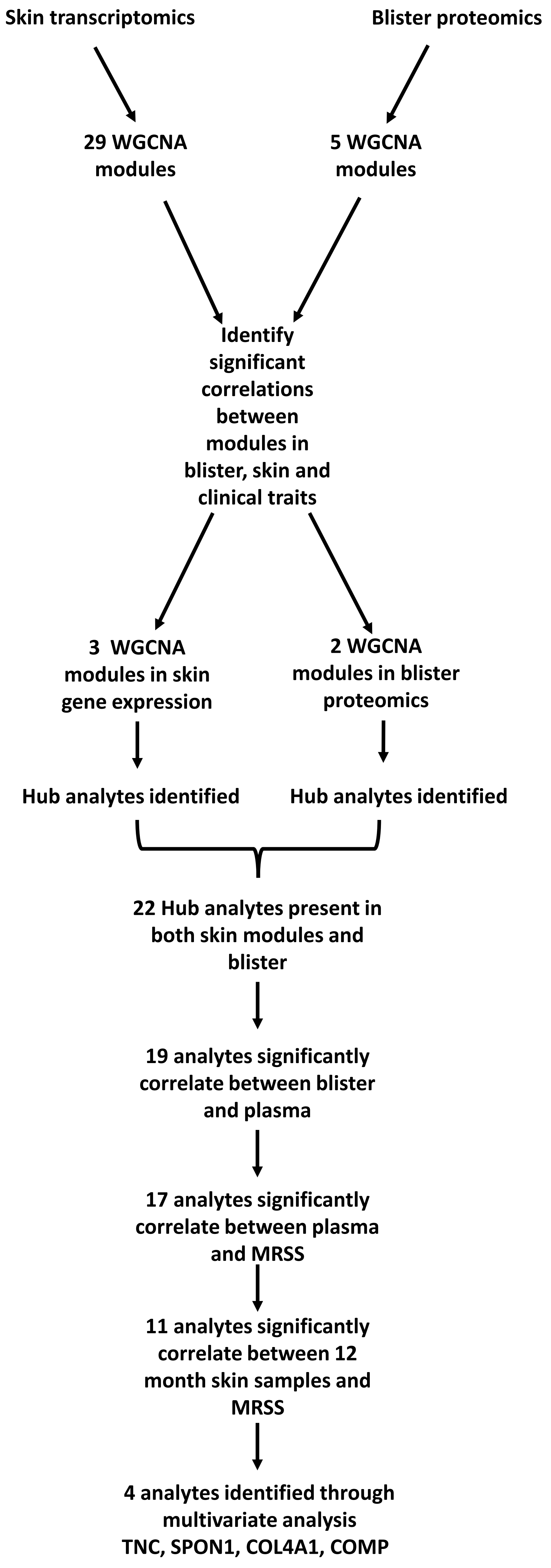

Supplementary figure 3: Soft thresholding power determination in WGCNA. Analysis of scale-free topology model fit index for soft threshold powers and mean connectivity of soft threshold powers in A) blister fluid proteomics, B) Skin transcriptomics, C) blood transcriptomics and D) plasma proteomics

A

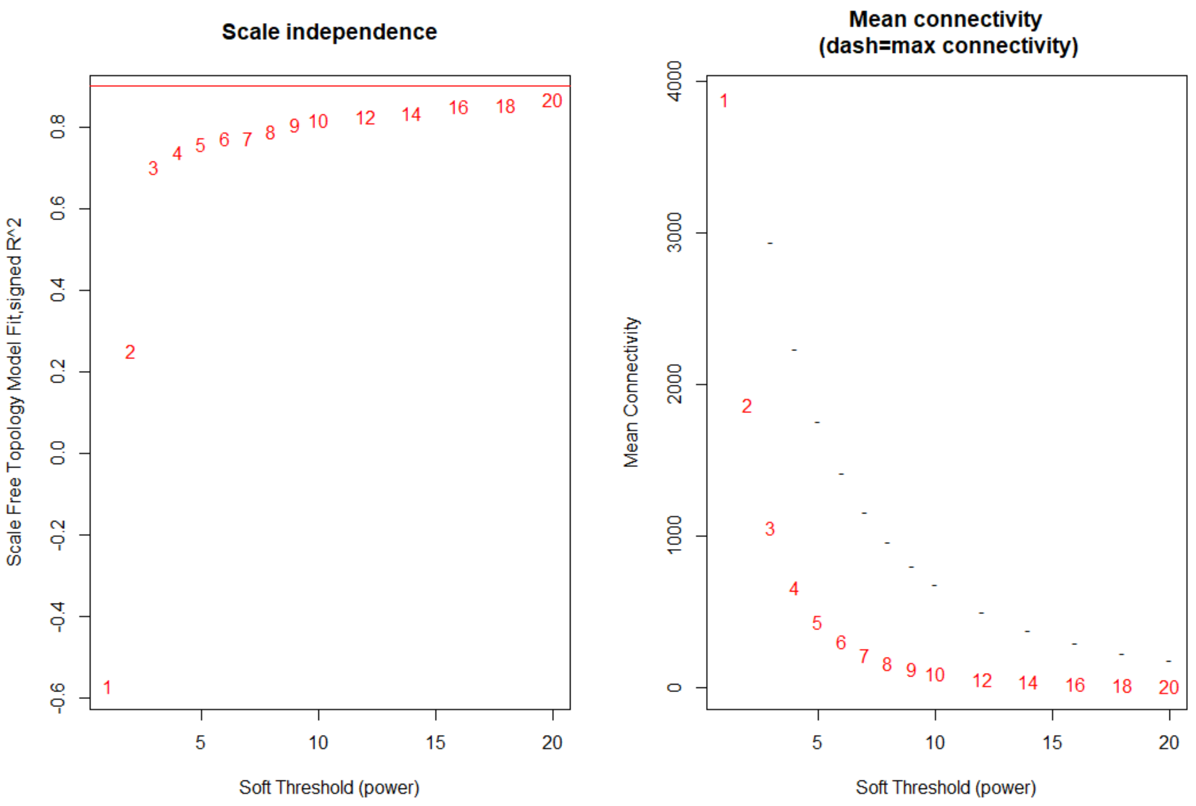

B

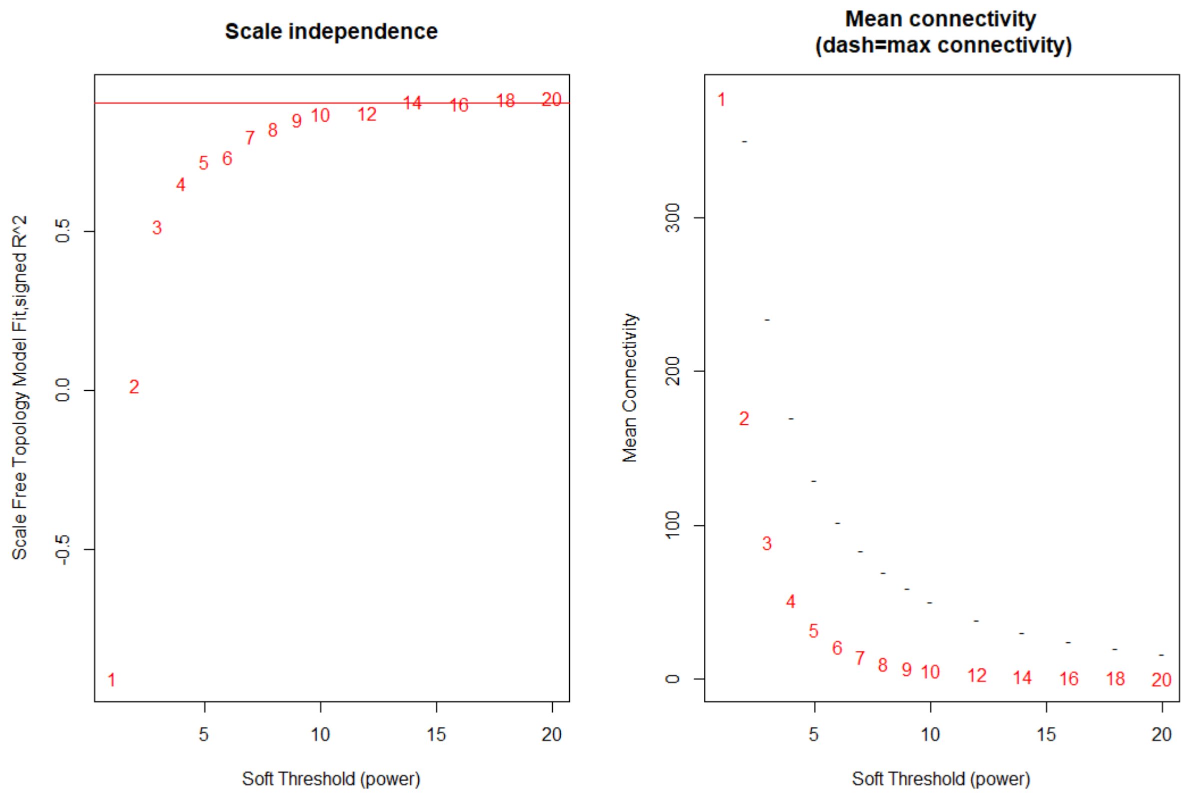

C

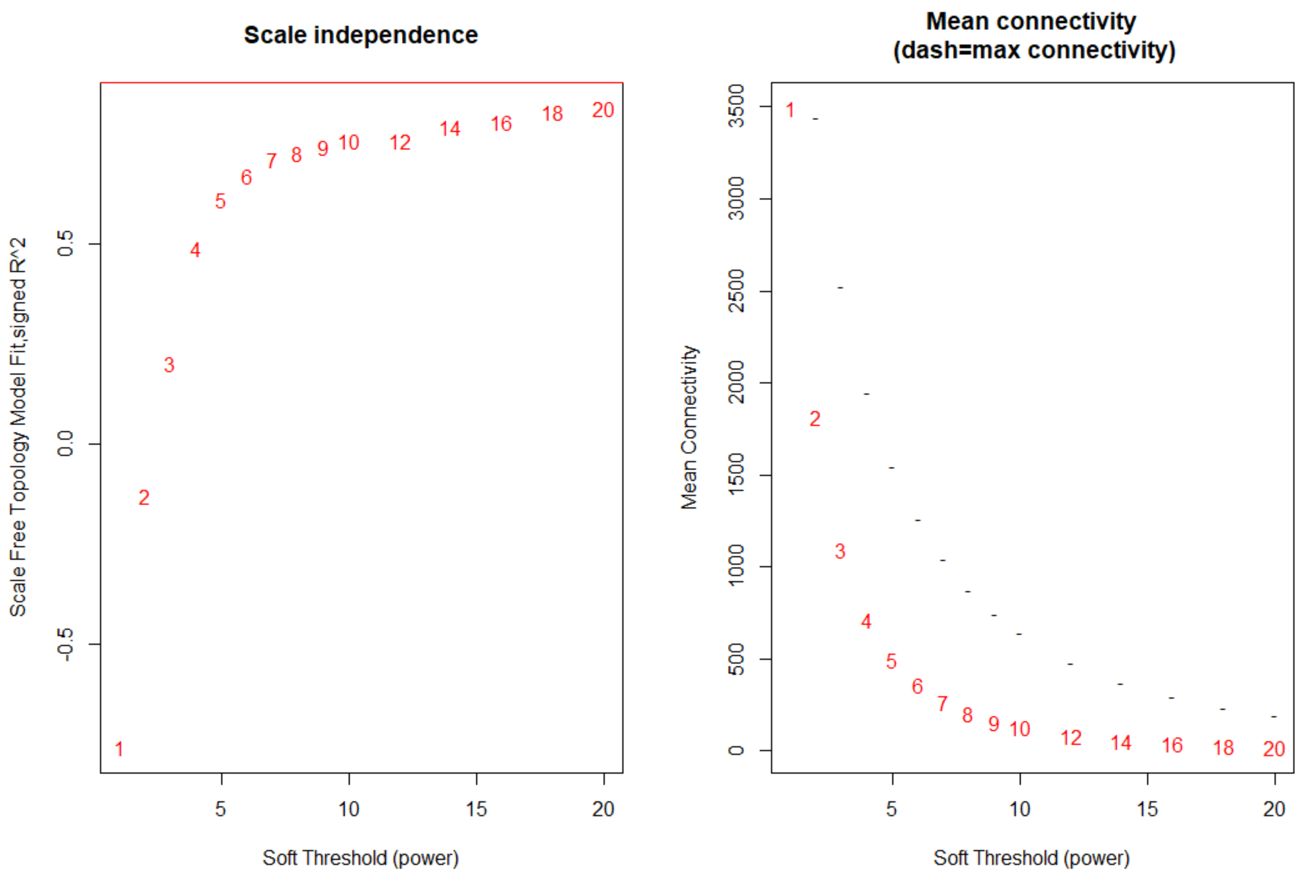

Plasma

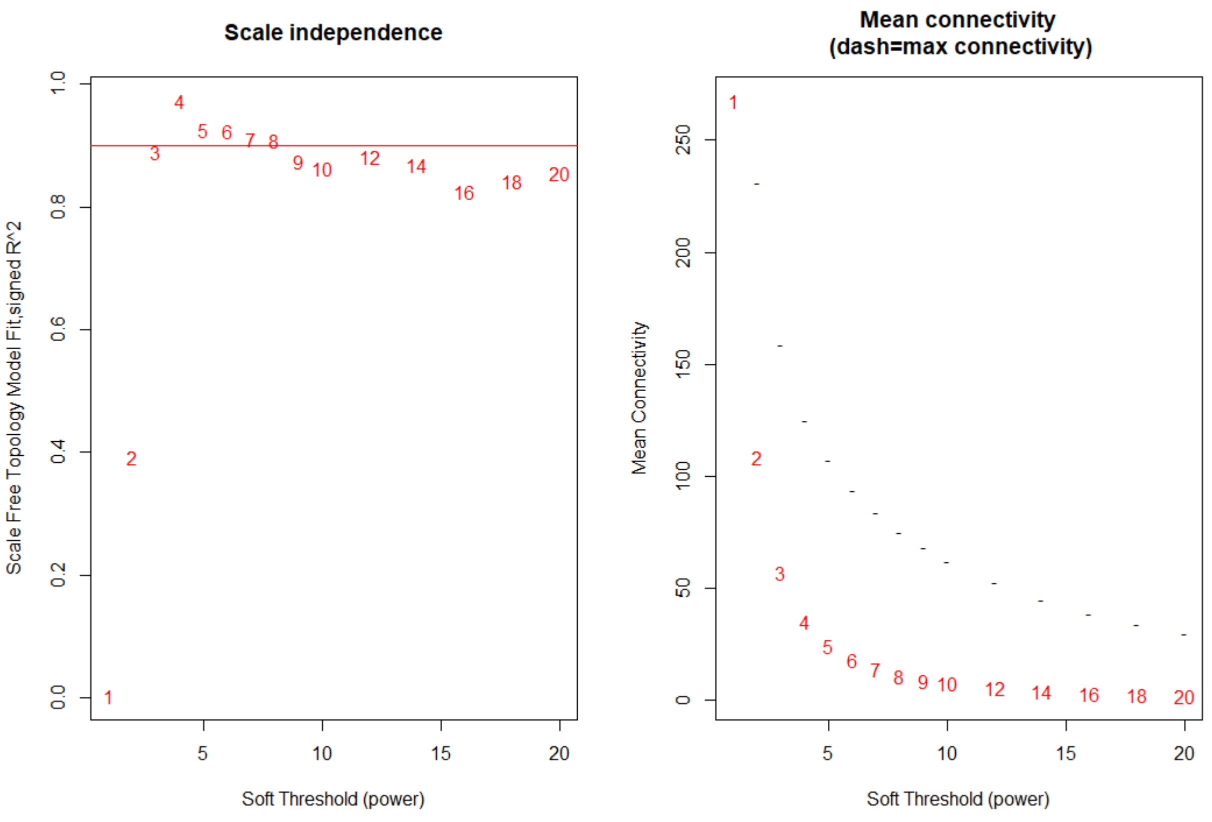

Supplementary figure 4: Identification of WGCNA modules in skin transcripts and blister proteomes. A) Module-trait relationships between module eigengenes of blood gene expression and clinical traits and in B) plasma proteomics. R^2 and p-value shown in each colour cell coded by the correlation between modules and traits (red indicates positive correlation). Blue indicates a positive correlation, and red negative correlation. Scl70 (anti-topoisomerase antibody), RNApol (anti-RNA polymerase III), HC (healthy control), CRP (C-reactive protein)

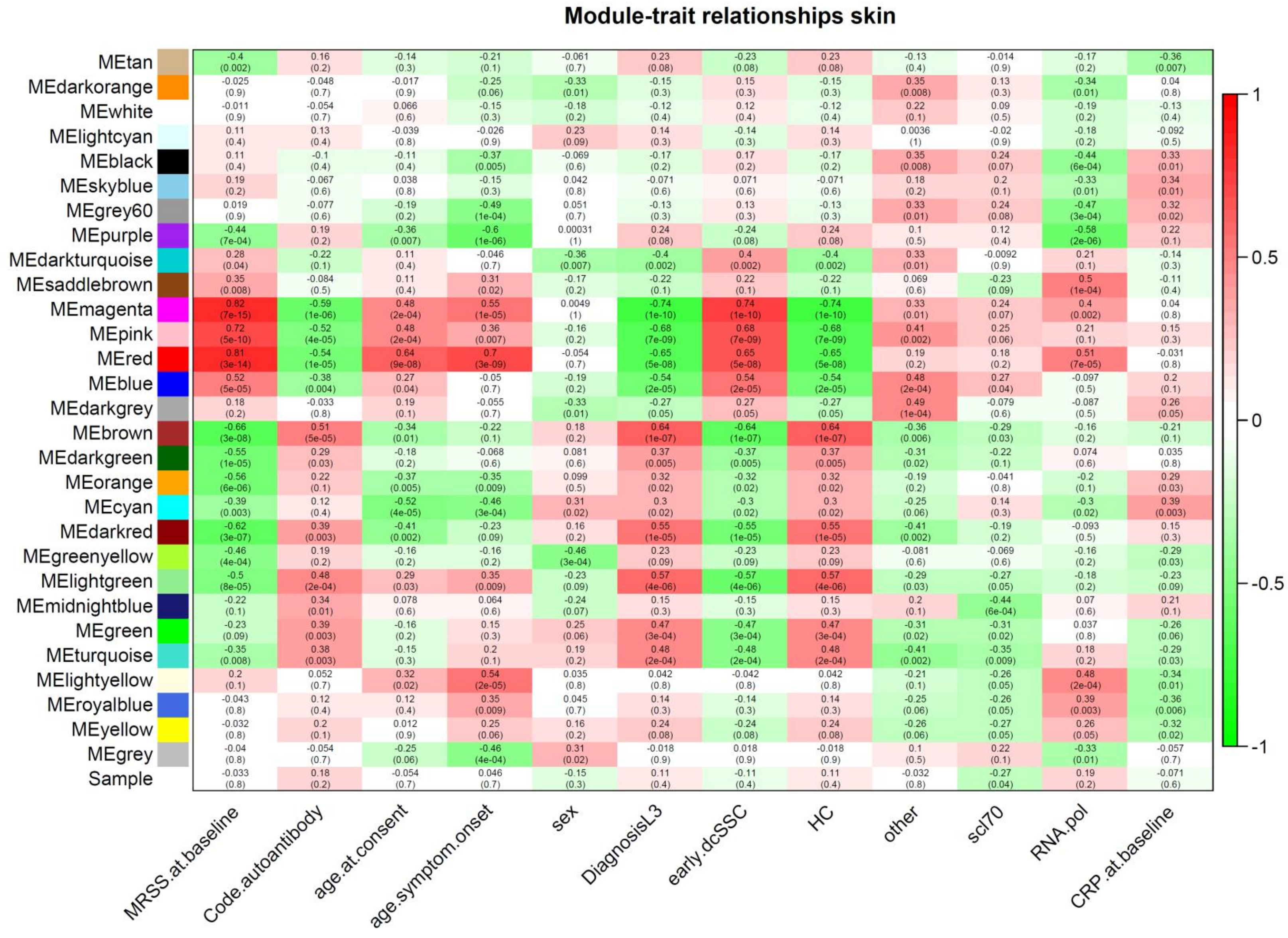

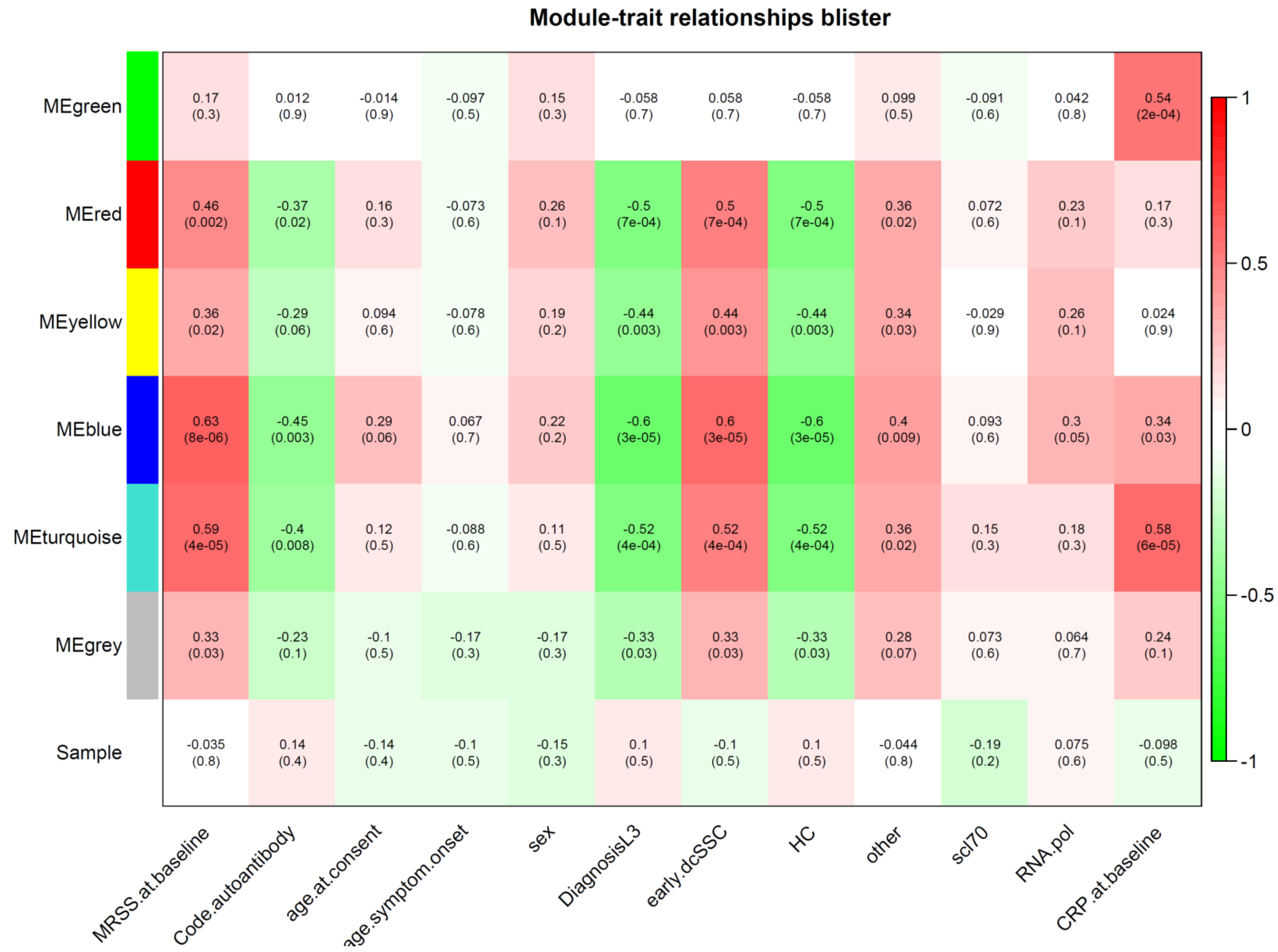

Supplementary figure 5: Gene significance and module membership in early dcSSc related modules. A+B) Distribution of average gene significance and standard error in each module related to early dcSSc in A) skin transcriptomics and B) blister proteomics. Scatter plot of module membership against gene significance to early dcSSc for C) blue blister module, E) turquoise blister module, D) red skin module, E) pink skin module, and F) magenta skin module. Correlation and p value included

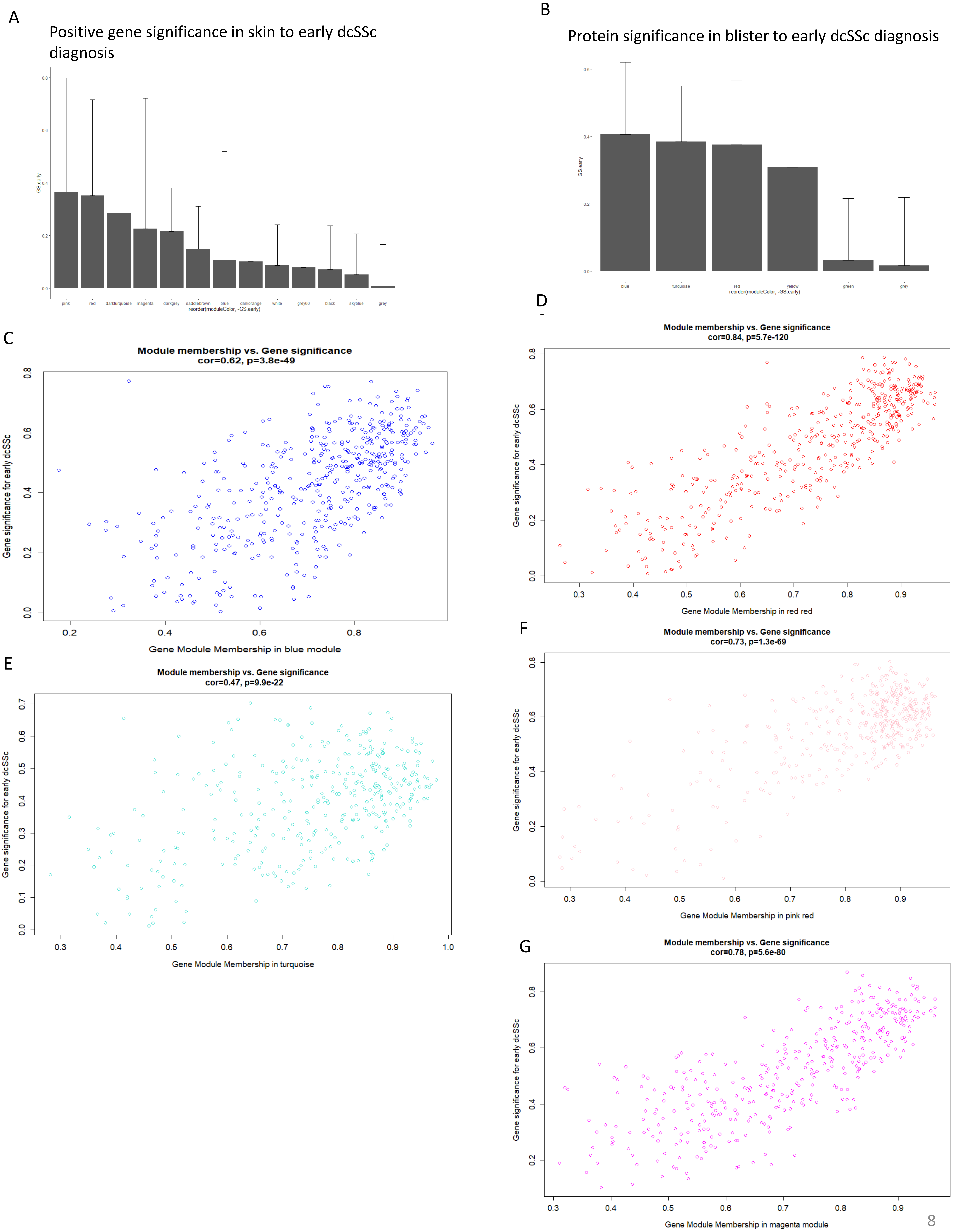

Supplementary figure 6: Dot plot functional annotation of early dcSSc related modules. dotplots for each significant module in skin (A, B, C) and blister (D, E). F) cnetplot showing interaction of proteins and biological concepts in the blue blister module.

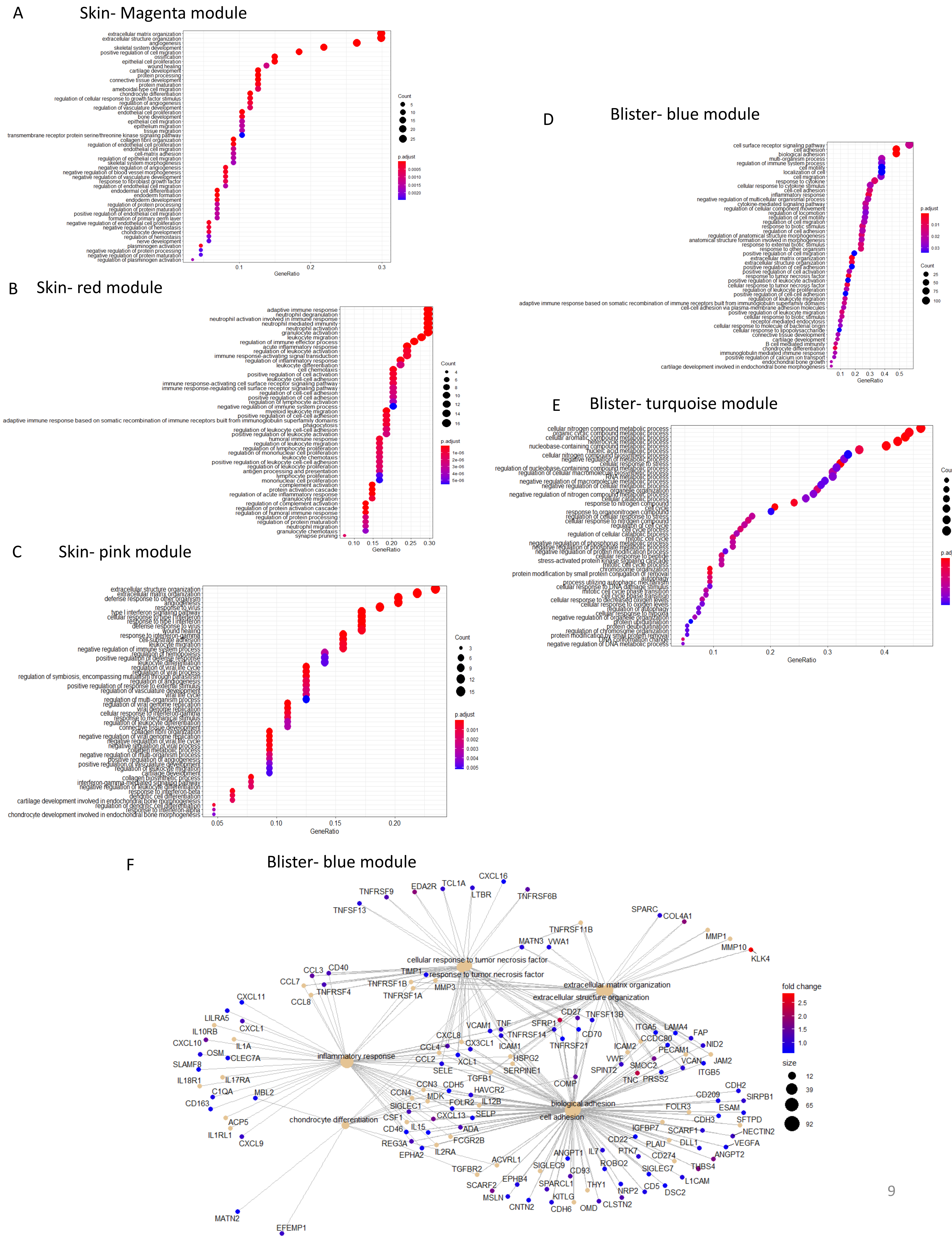

Supplementary figure 7: Cnetplots functional annotation of early dcSSc related modules and significant Hallmark pathway upregulation. for A) magenta skin module, B) pink skin module, C) red skin module, D) turquoise blister module. E) Cleveland plot showing normalised enrichment score for significant Hallmark pathways in each module of skin and blister.

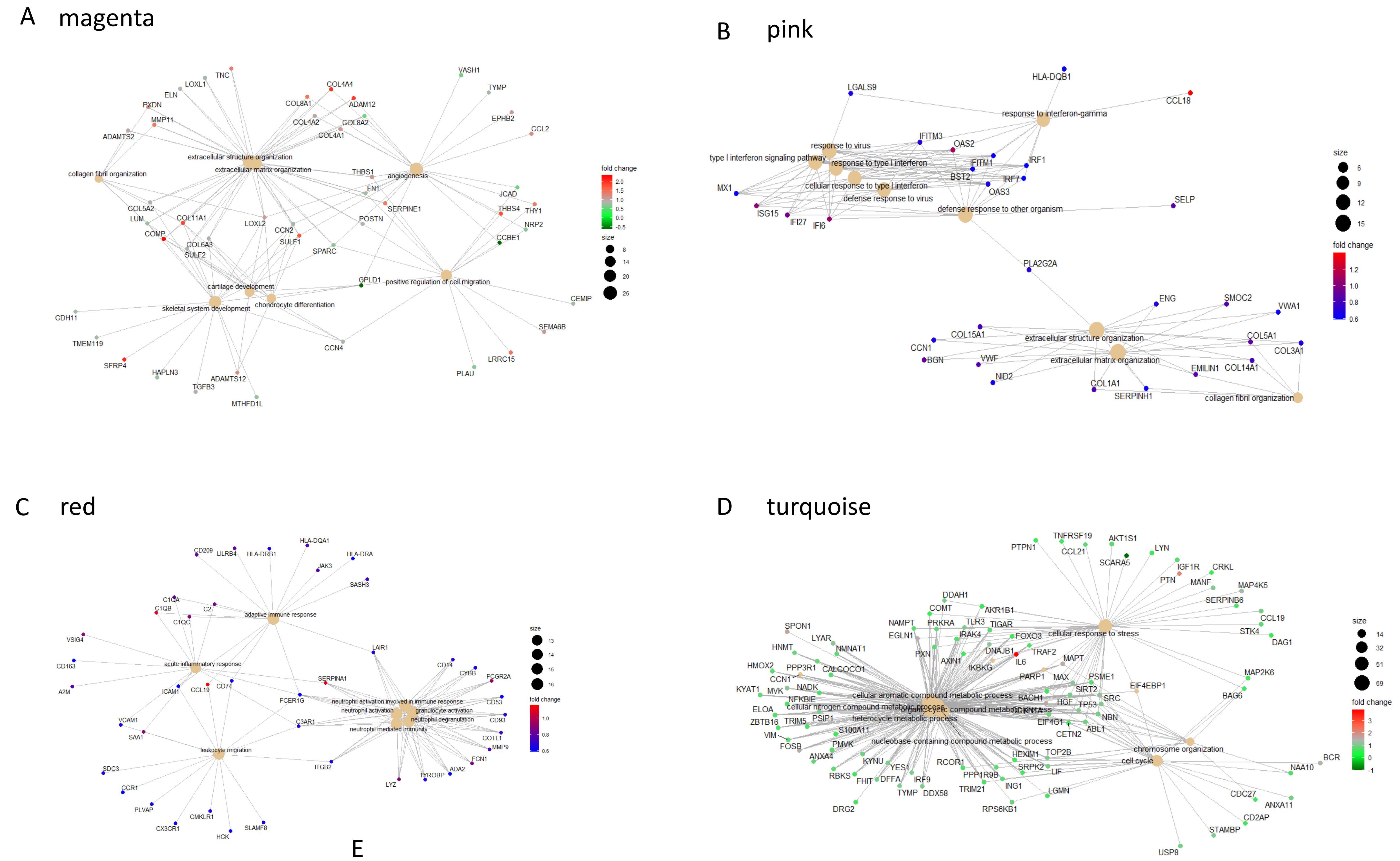

E

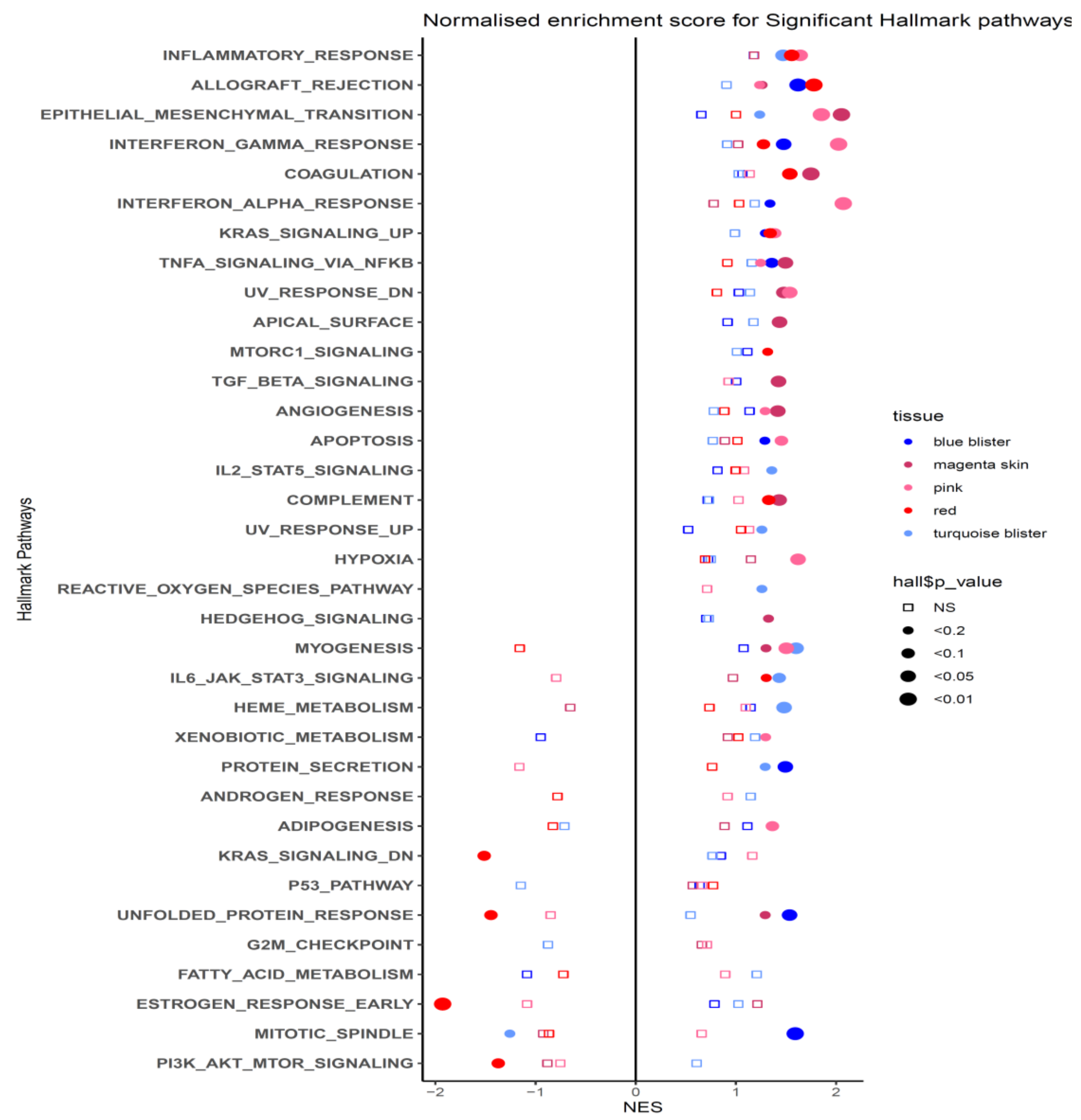

Supplementary figure 8: Correlations of 22 key analytes across tissue types. A) Correlation between 22 key analytes by gene expression and proteomics. Statistically significant correlations between blister and plasma concentrations in bold. B) Correlation between 19 key analytes and mRSS in each tissue modality. Statistically significant plasma proteomic correlations in bold. C) Correlation between skin gene expression in 12-month biopsies and mRSS in the 17 key analytes. Statistically significant corelations in bold. Abbreviations: mRSS (modified Rodnan skin score). ANGPT2 (angiopoietin 2), C1QA (complement C1q), CCN4 (cellular communication network factor 4), CD93 (cluster of differentiation 93), CLEC14A (C-Type Lectin Domain Containing 14A), COL4A1 (collagen 4A1), COMP (cartilage oligomatrix protein), CPXM1 (carboxypeptidase X, M14 Family member 1), EFEMP1 (EGF containing fibulin extracellular matrix protein 1), HAVCR2 (hepatitis A virus cellular receptor 2), IGFBP7 (insulin-like growth factor Binding protein 7), LTBP2 (Latent Transforming Growth Factor Beta Binding Protein 2), PGF (placental growth factor), SCARF2 (scavenger receptor Class F Member 2), SPON1 (spondin 1), THBS4 (thrombospondin 4), THY1 (Thy-1 Cell Surface Antigen), TNC (tenascin C), TNFRSF1B (TNF Receptor Superfamily Member 1B), VWA1 (von Willebrand Factor A Domain Containing 1).

A Key analytes showing correlation between gene and protein expression across the BIOPSY cohort

	blister vs skin		plasma vs blood		blister vs plasma		Skin vs plasma	
	r	p value	r	p value	r	p value		
**ANGPT2**	0·6144	**<0·0001**	0.0263	0.8498	0.4329	**0.002**	0.4546	**0.001**
**C1QA**	0·4653	**0·0005**	0.009	0.9488	0.448	**0.001**	-0.0185	0.896
**CCN4**	0·5264	**<0·0001**	0.087	0.5305	0.5194	**<0.0001**	0.4147	**0.003**
**CD93**	0·6592	**<0·0001**	0.2616	0.0563	0.6755	**<0.0001**	0.4574	**0.002**
**CLEC14A**	0·5149	**0·0001**	0.0705	0.6116	0.5161	**<0.0001**	0.2913	**0.040**
**COL4A1**	0·5670	**<0·0001**	0.1967	0.1536	0.4731	**0.001**	0.6986	**<0.0001**
**COMP**	0·7763	**<0·0001**	-0.1040	0.4530	0.5821	**<0.0001**	0.5951	**<0.0001**
CPXM1	0·8084	**<0·0001**	-0.0647	0.6413	0.2712	0.055	0.3729	**0.009**
**EFEMP1**	0·7147	**<0·0001**	0.1067	0.4415	0.5693	**<0.0001**	0.6876	**<0.0001**
**HAVCR2**	0·6524	**<0·0001**	0.1909	0.1663	0.5662	**<0.0001**	0.4653	**<0.0001**
**IGFBP7**	0·5168	**<0·0001**	0.1272	0.3583	0.5782	**<0.0001**	0.3242	**0.022**
**LTBP2**	0·7090	**<0·0001**	0.1827	0.1856	0.3849	**0.006**	0.4706	**0.001**
LYN	0·3863	**<0·0001**	0.1737	0.2085	0.0539	0.701	0.1646	0.262
NID2	0·6702	**<0·0001**	0.1775	0.1985	0.0553	0.726	-0.0325	0.856
**PGF**	0·6274	**<0·0001**	0.0690	0.6190	0.6206	**<0.0001**	0.4919	**0.001**
**SCARF2**	0·6694	**<0·0001**	0.1482	0.2841	0.6605	**<0.0001**	0.553	**<0.0001**
**SPON1**	0·7425	**<0·0001**	-0.0507	0.7153	0.4538	**0.001**	0.6293	**<0.0001**
**THBS4**	0·6809	**<0·0001**	0.0352	0.8002	0.5587	**<0.0001**	0.4274	**0.003**
**THY1**	0·6500	**<0·0001**	0.1312	0.3435	0.5069	**0.0001**	0.4578	**0.002**
**TNC**	0·6050	**<0·0001**	0.0488	0.7256	0.3217	**0.023**	0.3697	**0.009**
**TNFRSF1B**	0·5951	**<0·0001**	0.3806	**0.0048**	0.5517	**<0.0001**	0.4336	**0.002**
**VWA1**	0·4904	**0·0002**	0.1861	0.1773	0.2848	**0.045**	0.402	**0.005**

B Correlation of analytes from all BIOPSY samples with MRSS at baseline

	Skin transcriptomics		Blister proteomics		Blood transcriptomics		Plasma proteomics	
	r	p	r	p	r	p	r	p
**ANGPT2**	0.6895	**<0.0001**	0.6487	**<0.0001**	-0.032	0.8416	0.5	**0.0006**
**C1QA**	0.6049	**<0.0001**	0.5197	**<0.0001**	0.1361	0.6462	0.2903	**0.0353**
**CCN4**	0.4731	**<0.0001**	0.4976	**0.0001**	0.1061	0.6223	0.396	**0.0038**
**CD93**	0.7152	**<0.0001**	0.6194	**<0.0001**	0.2099	0.418	0.4344	**0.0016**
**CLEC14A**	0.62	**<0.0001**	0.5286	**<0.0001**	0.1626	0.5976	0.4561	**0.0012**
**COL4A1**	0.7495	**<0.0001**	0.4437	**0.0007**	0.2212	0.4554	0.5877	**0.0016**
**COMP**	0.7068	**<0.0001**	0.5672	**<0.0001**	0.0695	0.7301	0.4888	**0.001**
**EFEMP1**	0.6194	**<0.0001**	0.5153	**<0.0001**	0.1115	0.6370	0.4857	**0.0008**
**HAVCR2**	0.5381	**<0.0001**	0.4967	**0.0001**	0.2477	0.8227	0.4363	**0.0017**
IGFBP7	0.782	**<0.0001**	0.5084	**<0.0001**	0.1812	0.5404	0.1712	0.2115
LTBP2	0.5837	**<0.0001**	0.5851	**<0.0001**	0.0644	0.7182	0.2673	0.0512
**PGF**	0.6883	**<0.0001**	0.5173	**<0.0001**	0.0961	0.6422	0.4868	**0.0006**
**SCARF2**	0.7247	**<0.0001**	0.6093	**<0.0001**	-0.1217	0.6204	0.5386	**0.0008**
**SPON1**	0.6644	**<0.0001**	0.553	**<0.0001**	-0.1671	0.6007	0.4376	**0.0017**
**THBS4**	0.6284	**<0.0001**	0.5273	**<0.0001**	-0.0358	0.8645	0.4861	**0.005**
**THY1**	0.7654	**<0.0001**	0.5273	**<0.0001**	-0.0132	0.9157	0.4347	**0.0014**
**TNC**	0.6529	**<0.0001**	0.4442	**0.0007**	0.0818	0.6928	0.4097	**0.0028**
**TNFRSF1B**	0.6447	**<0.0001**	0.5099	**<0.0001**	0.1349	0.5837	0.3825	**0.0061**
**VWA1**	0.5998	**<0.0001**	0.4629	**0.0004**	0.0749	0.4275	0.339	**0.0135**

C Validation correlation of analytes from all BIOPSY samples in skin at 12 months with MRSS

	12month skin transcriptomics vs MRSS	
	r	p
**ANGPT2**	0.7457	**0.0006**
C1QA	0.2973	0.2465
**CCN4**	0.7543	**0.0005**
**CD93**	0.7211	**0.0011**
**CLEC14A**	0.4865	**0.0477**
**COL4A1**	0.586	**0.0134**
**COMP**	0.6204	**0.0079**
EFEMP1	0.4153	0.0974
HAVCR2	0.3539	0.1635
PGF	0.3735	0.1398
**SCARF2**	0.4853	**0.0483**
**SPON1**	0.6867	**0.0023**
**THBS4**	0.8292	**<0.0001**
**THY1**	0.6216	**0.0077**
**TNC**	0.484	**0.048**
TNFRSF1B	0.2383	0.357
VWA1	0.0909	0.7286

Supplementary figure 9: Correlations of key hub analytes. Correlation plots of plasma NPX values against mRSS for A) ANGPT2, B) CCN4, C) CD93, D) CLEC14A, E) EPEMP1, F) THBS14, G) THY1 and H) SCARF2. Correlation coefficient (r) and pvalue shown. ANGPT2 (angiopoietin 2), CCN4 (cellular communication network factor 4), CD93 (cluster of differentiation 93), CLEC14A (C-Type Lectin Domain Containing 14A), EFEMP1 (EGF containing fibulin extracellular matrix protein 1), THBS4 (thrombospondin 4), THY1 (Thy-1 Cell Surface Antigen),

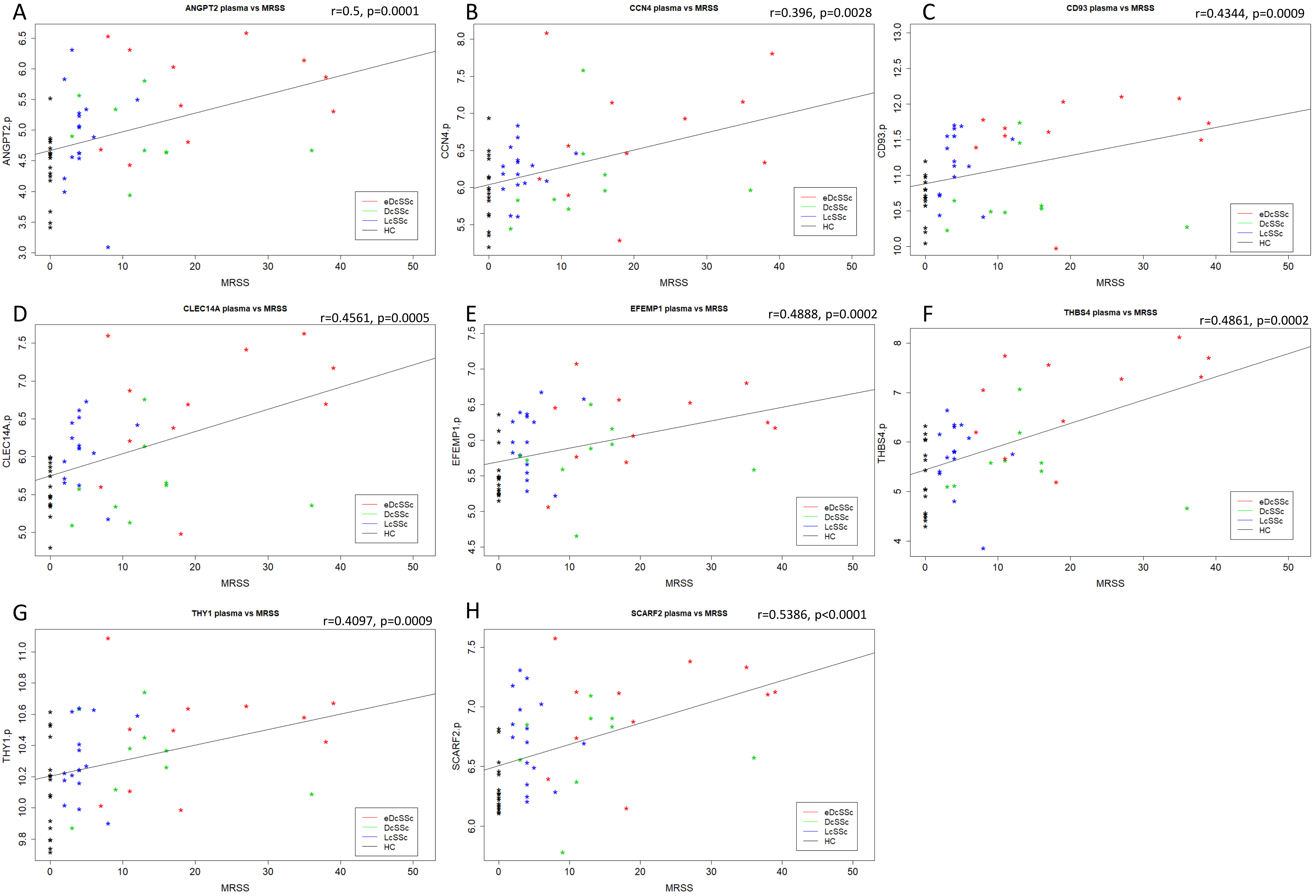

Supplementary Table 3: Mean expression level of 12 key hub analytes by disease subgroup. Confidence interval and p value from ANOVA included

	Mean (CI)				ANOVA
	HC	early dcSSc	late dcSSc	lcSSc	p value
ANGPT2	4.433 (4.101-4.765)	5.649 (4.798-6.501)	4.961 (4.122-5.799)	4.893 (4.089-5.691)	0.0003
CCN4	5.962 (5.685-6.238)	6.712 (6.002-7.423)	6.171 (5.472-6.871)	6.228 (5.56-6.897)	0.011
CD93	10.685 (10.438-10.932)	11.589 (10.955-12.222)	10.953 (10.329-11.577)	11.213 (10.617-11.809)	0.0002
CLEC14A	5.594 (5.314-5.873)	6.665 (5.948-7.381)	5.858 (5.152-6.563)	6.106 (5.432-6.781)	0.0001
COL4A1	5.959 (5.641-6.277)	7.471 (6.654-8.287)	6.716 (5.912-7.52)	6.753 (5.985-7.521)	<0.0001
COMP	7.619 (7.33-7.908)	8.511 (7.768-9.253)	8.097 (7.366-8.828)	7.731 (7.032-8.429)	0.001
EFEMP1	5.523 (5.292-5.755)	6.225 (5.632-6.819)	5.747 (5.163-6.332)	5.978 (5.42-6.537)	0.002
SCARF2	6.33 (6.152-6.508)	6.995 (6.538-7.452)	6.746 (6.296-7.196)	6.731 (6.301-7.161)	0.0001
SPON1	0.859 (0.678-1.04)	1.45 (0.986-1.914)	1.074 (0.617-1.531)	0.984 (0.548-1.421)	0.001
THBS4	5.241 (4.843-5.64)	6.94 (5.918-7.963)	5.860 (4.854-6.867)	5.750 (4.789-6.712)	<0.0001
THY1	10.124 (9.982-10.267)	10.47 (10.105-10.836)	10.338 (9.979-10.698)	10.294 (9.951-10.638)	0.024
TNC	3.957 (3.646-4.267)	4.96 (4.162-5.756)	4.279 (3.494-5.064)	4.251 (3.501-5)	0.002

Supplementary figure 10: Box and whisker plots of plasma NPX values by disease subgroup of SSc, and Healthy controls for A) ANGPT2, B) CCN4, C) CD93, D) CLEC14A, E) COMP, F) COL4A1, G) EPEMP1, H) SPON1, I) SCARF2 J)THBS14, K) THY1 and L) TNC. ANOVA and Tukey post-hoc significant p values also displayed. ANGPT2 (angiopoietin 2), CCN4 (cellular communication network factor 4), CD93 (cluster of differentiation 93), CLEC14A (C-Type Lectin Domain Containing 14A), COL4A1 (collagen 4A1), COMP (cartilage oligomatrix protein), EFEMP1 (EGF containing fibulin extracellular matrix protein 1), SPON1 (spondin 1), SCARF2 (scavenger receptor Class F Member 2), THY1 (Thy-1 Cell Surface Antigen), TNC (tenascin C), TNFRSF1B (TNF Receptor Superfamily Member 1B).

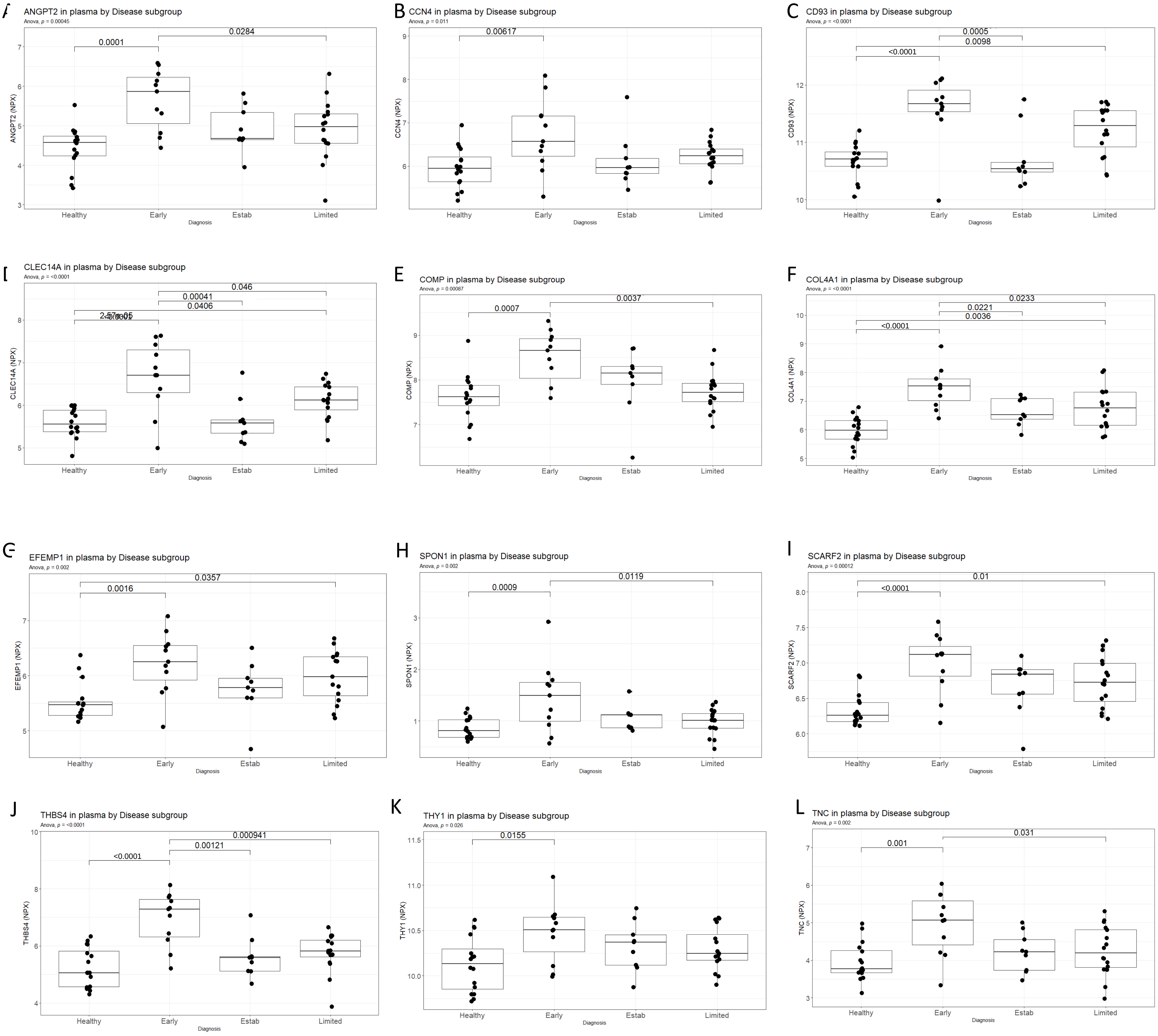

Supplementary figure 12: Identification of WGCNA modules in blood transcripts and plasma proteomes. A) Module-trait relationships between module eigengenes of blood gene expression and clinical traits and in B) plasma proteomics. R^2 and p-value shown in each colour cell coded by the correlation between modules and traits (red indicates positive correlation). C) Correlation matrix highlighting only significant correlations (p<0·05) between clinical traits, plasma fluid proteomics and blood transcriptomics. Blue indicates a positive correlation, and red negative correlation. Scl70 (anti-topoisomerase antibody), RNApol (anti-RNA polymerase III), HC (healthy control), CRP (C-reactive protein)

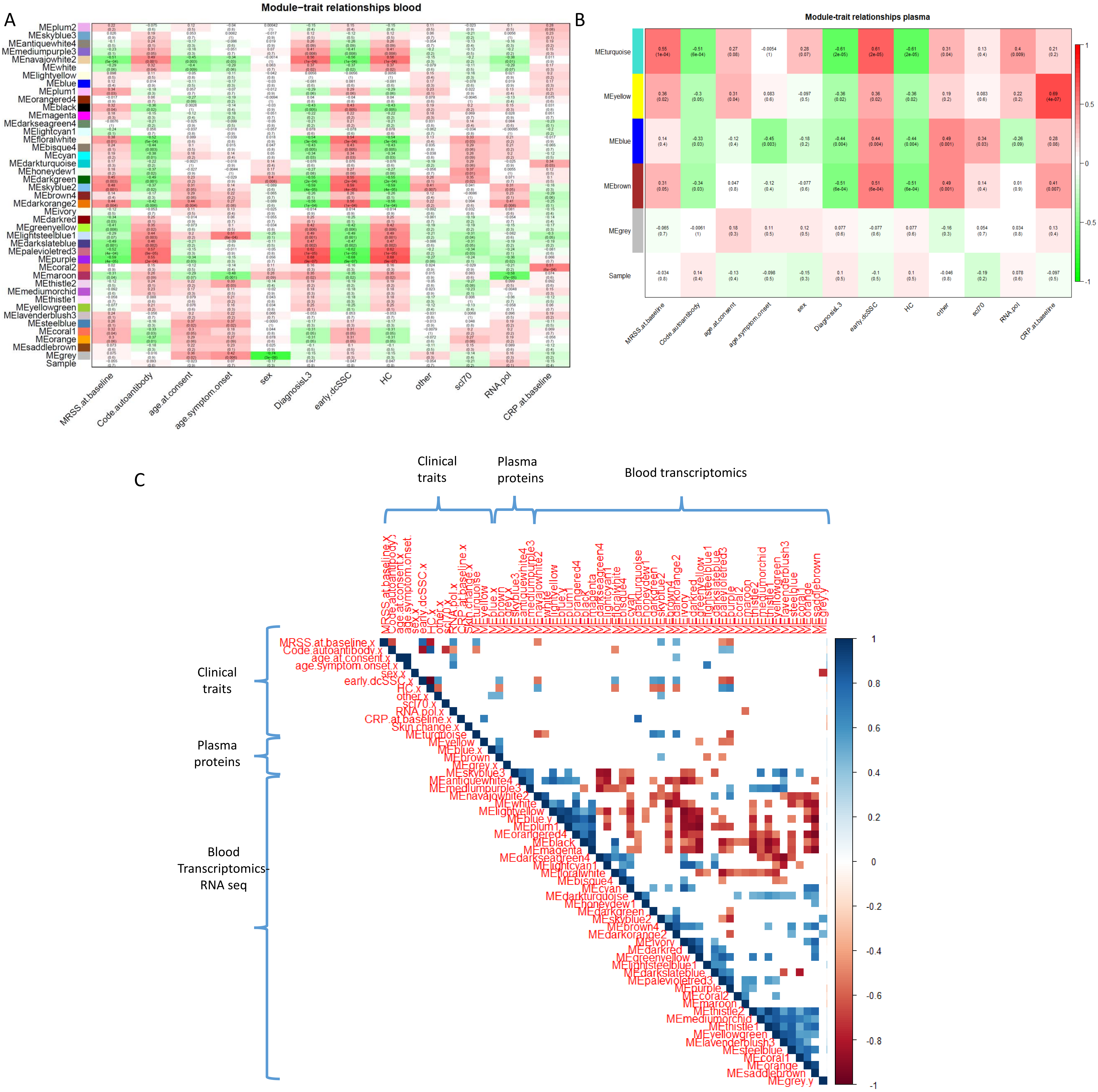

Supplement: Supplementary appendix [file mmc1.pdf]
